# NOP2-mediated 5-methylcytosine modification of APOL1 messenger RNA activates PI3K-Akt and facilitates clear cell renal cell carcinoma progression

**DOI:** 10.7150/ijbs.97503

**Published:** 2024-09-09

**Authors:** Junjie Tian, Jianguo Gao, Cheng Cheng, Zhijie Xu, Xiaoyi Chen, Yunfei Wu, Guanghou Fu, Baiye Jin

**Affiliations:** 1 Department of Reproductive Endocrinology, Women's Hospital, Zhejiang University School of Medicine, Hangzhou, China.; 2 Department of Urology, The First Affiliated Hospital, Zhejiang University School of Medicine, Hangzhou 310003, China.; 3 Zhejiang Engineering Research Center for Urinary Bladder Carcinoma Innovation Diagnosis and Treatment, Hangzhou 310003, China.

**Keywords:** 5-methylcytosine, clear cell renal cell carcinoma, NOP2, APOL1, clinical prognosis

## Abstract

**Background:** By regulating the functions of multiple RNAs, 5-methylcytosine (m^5^C) RNA methylation, particularly mediated by NOP2, is involved in tumorigenesis and developments. However, the specific functions and potential mechanisms of m^5^C, especially involving NOP2, in clear-cell renal cell carcinoma (ccRCC), remain unclear.

**Methods:** NOP2 expression in cell lines and patient tissues was detected using western blotting, quantitative real-time polymerase chain reaction (RT-qPCR), and immunohistochemistry. The biological effects of NOP2 on ccRCC cells were investigated through a series of *in vitro* and *in vivo* experiments. To explore the potential regulatory mechanisms by which NOP2 affects ccRCC progression, m^5^C bisulfite sequencing, RNA-sequencing, RNA immunoprecipitation and methylated RNA immunoprecipitation (RIP/MeRIP) RT-qPCR assay, luciferase reporter assay, RNA stability assay, and bioinformatic analysis were performed.

**Results:** NOP2 expression was significantly upregulated in ccRCC tissues and was associated with poor prognosis. Moreover, loss-of-function and gain-of-function assays demonstrated that NOP2 altered ccRCC cell proliferation, migration, and invasion. Mechanistically, NOP2 stimulated m^5^C modification of apolipoprotein L1 (APOL1) mRNA, and m^5^C reader YBX1 stabilized APOL1 mRNA through recognizing and binding to m^5^C site in the 3′-untranslated regions. Silencing APOL1 expression inhibited ccRCC cell proliferation *in vitro* and tumor formation *in vivo*. Furthermore, NOP2/APOL1 affected ccRCC progression via the PI3K-Akt signaling pathway.

**Conclusion:** NOP2 functions as an oncogene in ccRCC by promoting tumor progression through the m^5^C-dependent stabilization of APOL1, which in turn regulates the PI3K-Akt signaling pathway, suggesting a potential therapeutic target for ccRCC.

## Introduction

Renal cell carcinoma (RCC) is a common urological malignancy with an increasing global incidence 1. Clear cell renal cell carcinoma (ccRCC) is the most prevalent subtype, accounting for almost 80% of all primary kidney tumors. Given the absence of clinical symptoms during the early stages, approximately 25-30% of patients with ccRCC present with distant metastasis at primary diagnosis. The relative 5-year survival rate of patients with ccRCC decreases from 91% to less than 10% once the primary tumor metastasizes or becomes aggressive 2. Despite advances in chemotherapeutic drugs, resistance to chemotherapy remains a major challenge in ccRCC treatment 3. In recent years, although the clinical application of targeted therapy and immunotherapy has improved the overall survival (OS) of patients to a certain extent 4, many patients with advanced ccRCC do not exhibit favorable responses to these therapeutic approaches due to inter-individual differences in treatment response and drug resistance 5-7. Although the pathogenesis of ccRCC remains unclear, it may be related to genetic and chromosomal abnormalities, including gene fusion 5, 8 and aberrant DNA methylation 9, 10. Therefore, further research into the molecular mechanisms driving ccRCC progression and metastasis is imperative for developing innovative therapeutic strategies.

Recently, RNA epigenetics/epitranscriptomics has gained considerable attention 11-13. To date, more than 170 types of RNA modifications have been identified in messenger RNA (mRNA), ribosomal RNA (rRNA), and transfer RNA (tRNA) 14. Methylation is one of the main forms of RNA modification, and N6-methyladenosine (m^6^A) methylation is the most prevalent and reversible modification in eukaryotic RNA. Recently, another important RNA modification, 5-methylcytosine (m^5^C), was identified in tRNAs and rRNAs 15, 16. Moreover, m^5^C modification is enriched in CG-rich regions and regions immediately downstream of the translation initiation site of mRNAs 17, 18, which is associated with mRNA metabolism, including nuclear export, degradation, and translation. In addition, m^5^C modification is associated with the development and progression of various tumor and non-tumor diseases 19. NOP2 (also known as NSUN1, NOL1, p120, or NOP120), a member of the NOP2/NSUN RNA methyltransferase family, contains an RNA-binding domain and an RNA methyltransferase domain. NOP2 plays an important regulatory role in a variety of biological functions, including cellular proliferation 20, migration 21, differentiation 22, and tumorigenesis through m^5^C-dependent mechanisms 21, 23, 24. Moreover, immunohistochemical analysis revealed that NOP2 is upregulated in various cancers, including breast cancer 25, colorectal cancer 26, hepatocellular carcinoma 21, lung adenocarcinoma 27, 28, and oral carcinoma 29. Given its potential oncogenic role 30, NOP2 is a key regulator of m^5^C RNA methylation that affects tumorigenesis and development. However, the underlying regulatory mechanisms and distribution of NOP2 in human RCC remain unclear.

Our findings revealed that NOP2 is significantly upregulated in ccRCC cells and tissues and is associated with poor prognosis in patients with ccRCC. Loss-of-function and gain-of-function assays demonstrated that NOP2 alters ccRCC cell proliferation both *in vitro* and *in vivo*. Furthermore, APOL1 is a potential downstream gene regulated by NOP2 in ccRCC. Mechanistically, NOP2 stimulated m^5^C modification of apolipoprotein L1 (APOL1) mRNA, and the m^5^C reader YBX1 stabilized APOL1 mRNA through recognizing and binding to the m^5^C site in 3′-untranslated regions, which subsequently affected ccRCC progression via the PI3K-Akt signaling pathway. Taken together, these findings suggest that NOP2-mediated m^5^C methylation of APOL1 mRNA regulates the PI3K/Akt signaling pathway and may serve as a novel mechanism for ccRCC progression. The findings of this study are presented as a graphical summary (Fig. 8).

## Results

### Expression landscape and clinical relevance of m^5^C regulators in ccRCC

**Figure 1:**
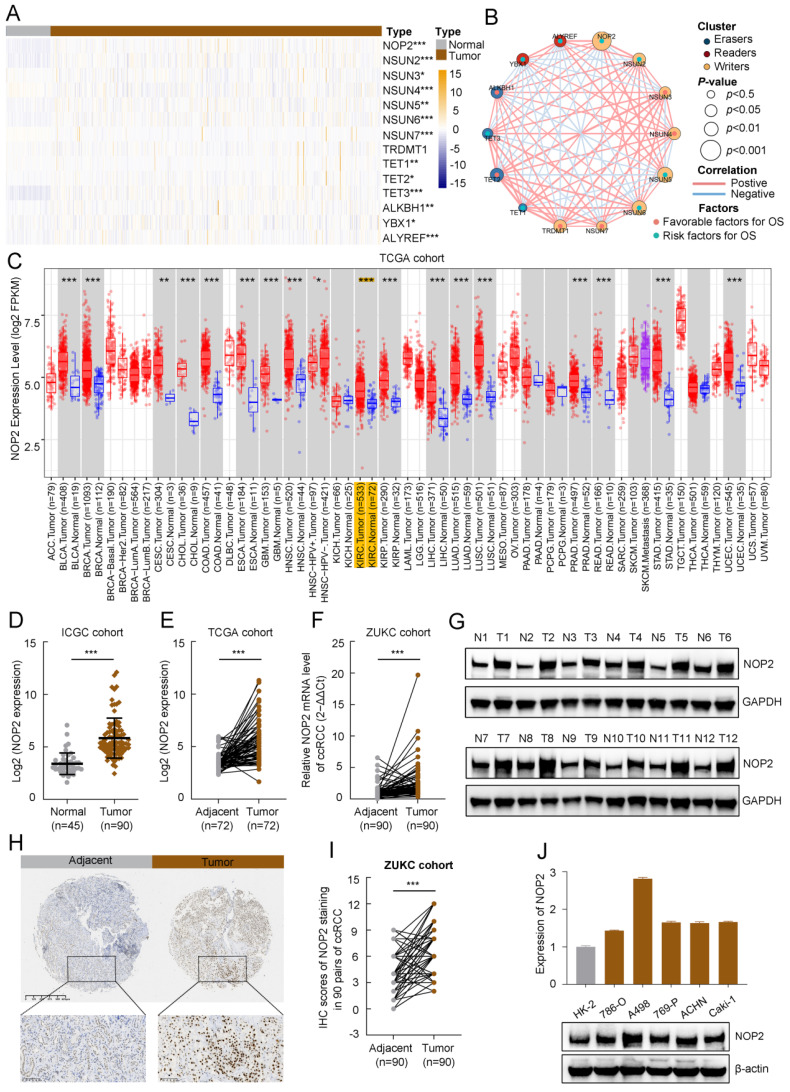
** The expression level of NOP2 in ccRCC tissue and cell lines.** (A)Heatmap visualized the expression of m^5^C regulators in TCGA-KIRC cohort. Yellow and blue regions represented higher and lower expression level, respectively. (**B**) Landscape of interactions network between m^5^C regulators in ccRCC. The circle size represented impact of each regulator on survival prognosis, as calculated by log-rank test. Connecting lines represented m^5^C regulators interactions. The red line represented positive correlation, the blue line represented negative correlation, and the line thickness indicated correlation strength (the wider the line, the stronger the correlation). The regulator clusters of Writers, Readers and Erasers were marked yellow, red, and blue, respectively. (**C**) Expression of NOP2 across various cancers in TCGA database. Yellow background represented KIRC patients. NOP2 was significantly upregulated in ccRCC tissues compared to the counterpart peritumoral normal renal tissues from the ICGC database (**D**) and paired normal tissues from the TCGA database (**E**). (**F**) Relative expression of NOP2 mRNA in 90 pairs of ccRCC tissues and their paired normal adjacent tissues from ZUKC cohort. (**G**) The expression of NOP2 protein was detected by Western blotting in 12 paired ccRCC tissues and adjacent normal kidney tissues. T: Tumor tissues, N: Adjacent normal tissues. (**H**) representative IHC images of NOP2 staining in ccRCC tumor or adjacent tissues from ZUKC tissue microarray. (**I**) IHC scores of 90 pairs of ccRCC tissues in ZUKC cohort according to NOP2 staining. (**J**) The mRNA and protein levels of NOP2 were detected in normal human renal epithelial cell line (HK-2) and RCC cell lines (786-O, A498, 769-P, ACHN and Caki-1) by RT-qPCR and Western blotting. Data were displayed as mean ± SD. Differences were considered significant at *P* < 0.05 (* *P* < 0.05, ** *P* < 0.01, *** *P* < 0.001).

To evaluate the expression profiles of m^5^C regulators, 14 m^5^C-related gene transcriptomic profiles were obtained from The Cancer Genome Atlas Kidney renal clear cell carcinoma (TCGA-KIRC). The overall expression levels of the m^5^C regulators are shown in a heatmap (Fig. 1A). The m^5^C regulators were significantly differentially expressed between tumor and normal samples from ccRCC patients. Furthermore, the m^5^C regulator network described the landscape of their interactions and their impact on the overall survival of ccRCC patients (Fig. 1B). These findings demonstrated that NOP2 was closely related to other m^5^C regulators and NOP2 is a risk factor significantly affecting the prognosis of ccRCC patients.

### Upregulated NOP2 expression in tumor samples is associated with poor prognosis in ccRCC patients

TCGA pan-cancer data showed that NOP2 is overexpressed in various types of human cancers (Fig. 1C), suggesting that NOP2 may function as a common oncogene involved in tumorigenesis and progression, including ccRCC. Additionally, NOP2 was upregulated in cancer cells (n = 90) than in control cells in the International Cancer Genome Consortium (ICGC)-ccRCC cohort (n = 45) (Fig. 1D) and the paired TCGA-KIRC cohort (n = 72) (Fig. 1E). Moreover, NOP2 overexpression at the mRNA level was observed in the paired ZUKC validation cohort (n = 90) (Fig. 1F). Consistent with these findings, the protein level of NOP2 was remarkably higher in 11/12 (92%) human ccRCC tissues than in paired normal kidney tissues by western blotting (Fig. 1G). Immunohistochemical analyses further validated these results in the ZUKC cohort (Fig. 1H, I).

Moreover, we analyzed the correlation between NOP2 expression and clinicopathological features in the TCGA-KIRC cohort. The protein expression of NOP2 in the ccRCC cohort was significantly correlated with clinicopathological features such as histologic grade, pathologic stage, and TNM stage (Supplementary Table S1). The association between NOP2 expression and clinicopathological characteristics was assessed in the ZUKC cohort (Supplementary Table S2). The final results showed significant correlations between NOP2 expression and older age, histologic grade, T stage, N stage, and tumor size. Finally, the difference in NOP2 expression was validated using RT-qPCR and Western blot analysis in a normal human renal epithelial cell line and several RCC cell lines (Fig. 1J). These results indicate that NOP2 may be a potential oncogene involved in ccRCC tumorigenesis and progression.

In addition, survival analyses showed that ccRCC patients with increased NOP2 expression had poorer OS (Supplementary Fig. S1A), progression-free survival (PFS) (Supplementary Fig. S1B), and disease-specific survival (DSS) (Supplementary Fig. S1C). Univariate and multivariable Cox regression analyses revealed that NOP2 was an independent and significant prognostic factor for OS in patients with ccRCC (Supplementary Fig. S1D, E). We further evaluated the prognostic value of NOP2 expression in combination with the clinicopathological characteristics. The 3-, 5-, and 8-year nomograms predicting the OS of ccRCC patients were established based on age, histologic grade, pathologic stage, and NOP2 expression level (Supplementary Fig. S1F, G), according to the results of the multivariable Cox regression analysis in the TCGA-KIRC cohort. Taken together, these results revealed that NOP2 was an independent prognostic factor, and upregulated NOP2 expression is associated with poor prognosis in ccRCC patients.

### NOP2 promoted human ccRCC cell proliferation and tumor growth in mice

**Figure 2:**
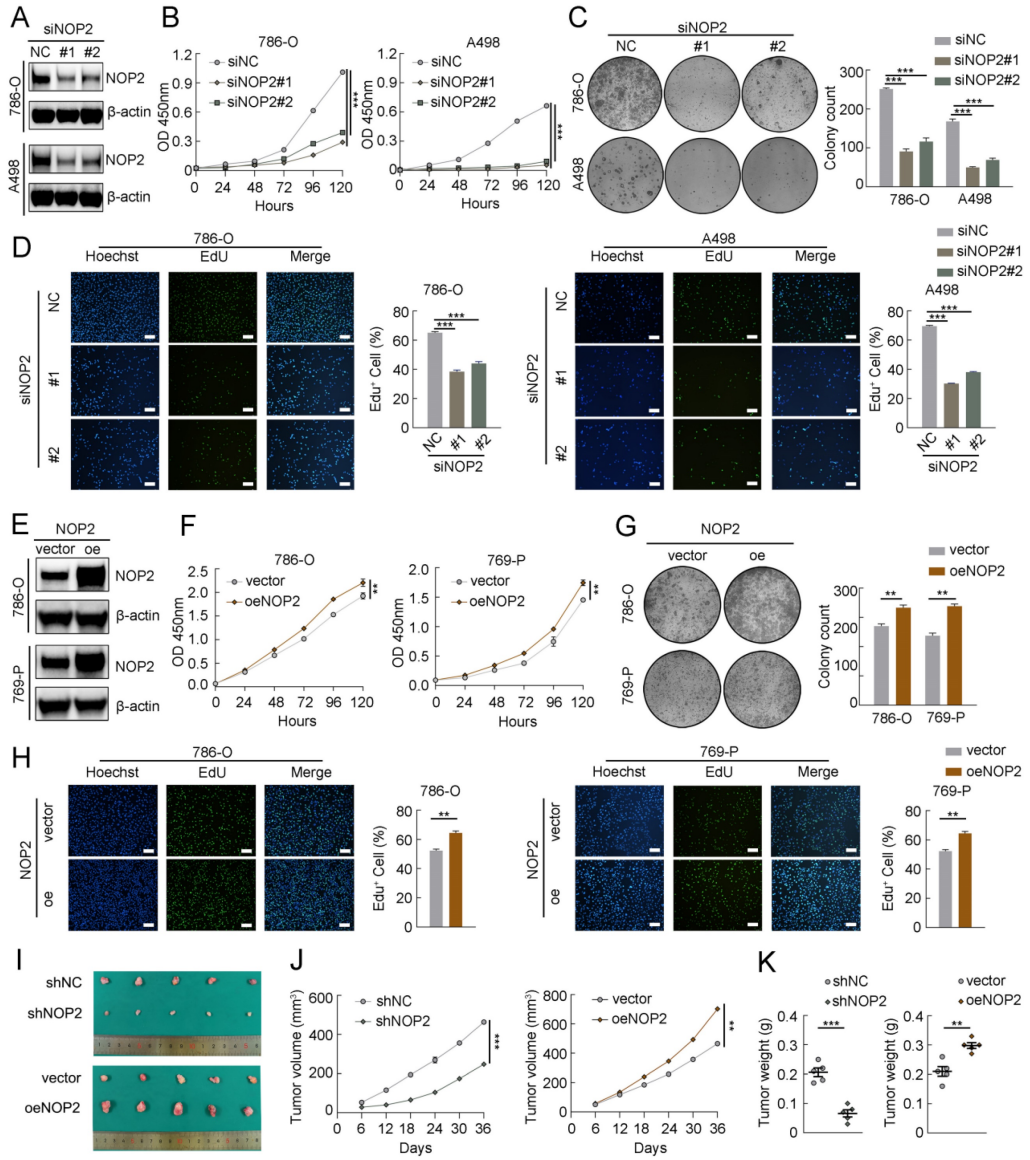
** NOP2 promoted ccRCC proliferation *in vitro* and* in vivo*.** (A)Western blotting analysis of NOP2 knockdown efficiency in 786-O and A498 cells. (**B-D**) The proliferation of ccRCC cells under silenced NOP2 was detected via CCK-8 (**B**), colony-formation (**C**), and EdU assays (**D**). (**E**) Western blotting analysis of NOP2 overexpression efficiency in 786-O and 769-P cells. (**F-H**) The proliferation of ccRCC cells under overexpression of NOP2 was detected via CCK-8 (**F**), colony-formation (**G**), and EdU assays (**H**). (**I-K**) Tumor growth nodules (**I**) of stable NOP2 knockdown and overexpression 786-O cells (or negative control) in the xenograft mouse model were shown, followed by the generation of tumor curve (**J**) and tumor weight records (**K**). Scale bars, 50 µm. Data were displayed as mean ± SD. Differences were considered significant at *P* < 0.05 (** *P* < 0.01, *** *P* < 0.001).

To explore the malignant behavior of NOP2 in ccRCC cells, we used small interfering RNAs (siRNAs) to knock down its expression in 786-O and A498 cells. NOP2 expression significantly decreased, as determined by western blotting (Fig. 2A). Subsequently, the Cell Counting Kit-8 (CCK-8), 5-ethynyl-2′-deoxyuridine (EdU), and colony formation assays showed that NOP2 downregulation substantially reduced cell proliferation and clonogenic ability (Fig. 2B-D). In contrast, NOP2 overexpression significantly promoted cell proliferation and clonogenic ability in these cell lines compared to that in controls (Fig. 2E-H). Simultaneously, rescue assays were conducted using cell lines with stable knockdown and re-overexpression of NOP2. Notably, re-overexpression of NOP2 in the knockdown cells completely rescued the defects in cell proliferation ability (Supplementary Fig. S2A-C). Furthermore, animal model experiments were conducted to verify the roles of NOP2 *in vivo*, which showed that NOP2 depletion suppressed tumor growth, as reflected by the tumor size and weight compared with those of tumors formed from wild-type (Wt) 786-O cells, whereas overexpression of NOP2 significantly promoted tumor growth in nude mice (Fig. 2I-K). Taken together, our results indicate that NOP2 promotes ccRCC cell proliferation *in vitro* and* in vivo*.

### NOP2 promoted *in vitro* migration and invasion and affected apoptosis in ccRCC cells

**Figure 3:**
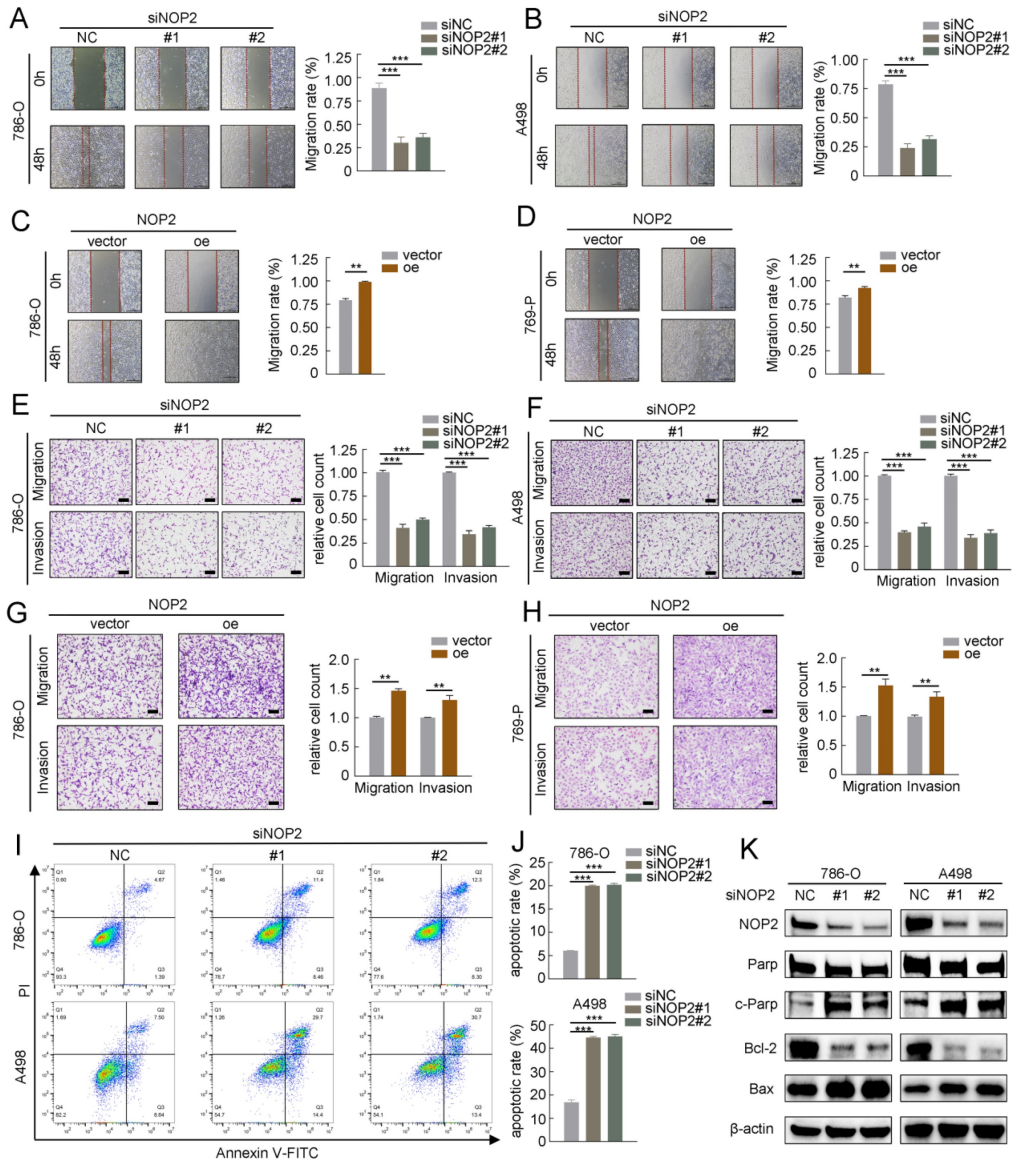
** NOP2 promoted *in vitro* migration and invasion and affected apoptosis of ccRCC cells.** Cell wound-healing assay (**A-D**), Transwell migration and invasion assay (**E-H**) revealed the effect of NOP2 knockdown or overexpression on ccRCC cells. Knockdown of NOP2 inducing apoptosis of ccRCC cells were detected by flow cytometry (**I**) and Western blotting assay (**J**). The corresponding quantitative analysis results were presented in the right panel. Scale bar, 50 μm. Data were displayed as mean ± SD. Differences were considered significant at *P* < 0.05 (** *P* < 0.01, *** *P* < 0.001).

To further investigate the role of NOP2 in ccRCC progression, we performed *in vitro* migration and invasion assays using ccRCC cells with knockdown or overexpression of NOP2. The migration and invasion rates of 786-O and A498 cells were abrogated by NOP2 knockdown via wound healing and transwell assays. Moreover, there was an increase in the migration and invasion abilities of 786-O and 769-P cells overexpressing NOP2 compared with those of control cells (Fig. 3A-H). Similarly, re-overexpression of NOP2 in knockdown cells completely restored their cell migration ability (Supplementary Fig. S2D-G). Subsequently, the effect of NOP2 on apoptosis was examined in ccRCC cells. Flow cytometry assay of 786-O and A498 cells revealed that NOP2 knockdown-induced apoptosis inhibited proliferation (Fig. 3I), thereby inhibiting cell progression. Moreover, alterations in cleaved PARP, Bcl-2, and Bax protein expression levels were observed, confirming the apoptosis results (Fig. 3J).

### NOP2-mediated m^5^C modification of APOL1 mRNA maintained its YBX1-dependent stability

**Figure 4:**
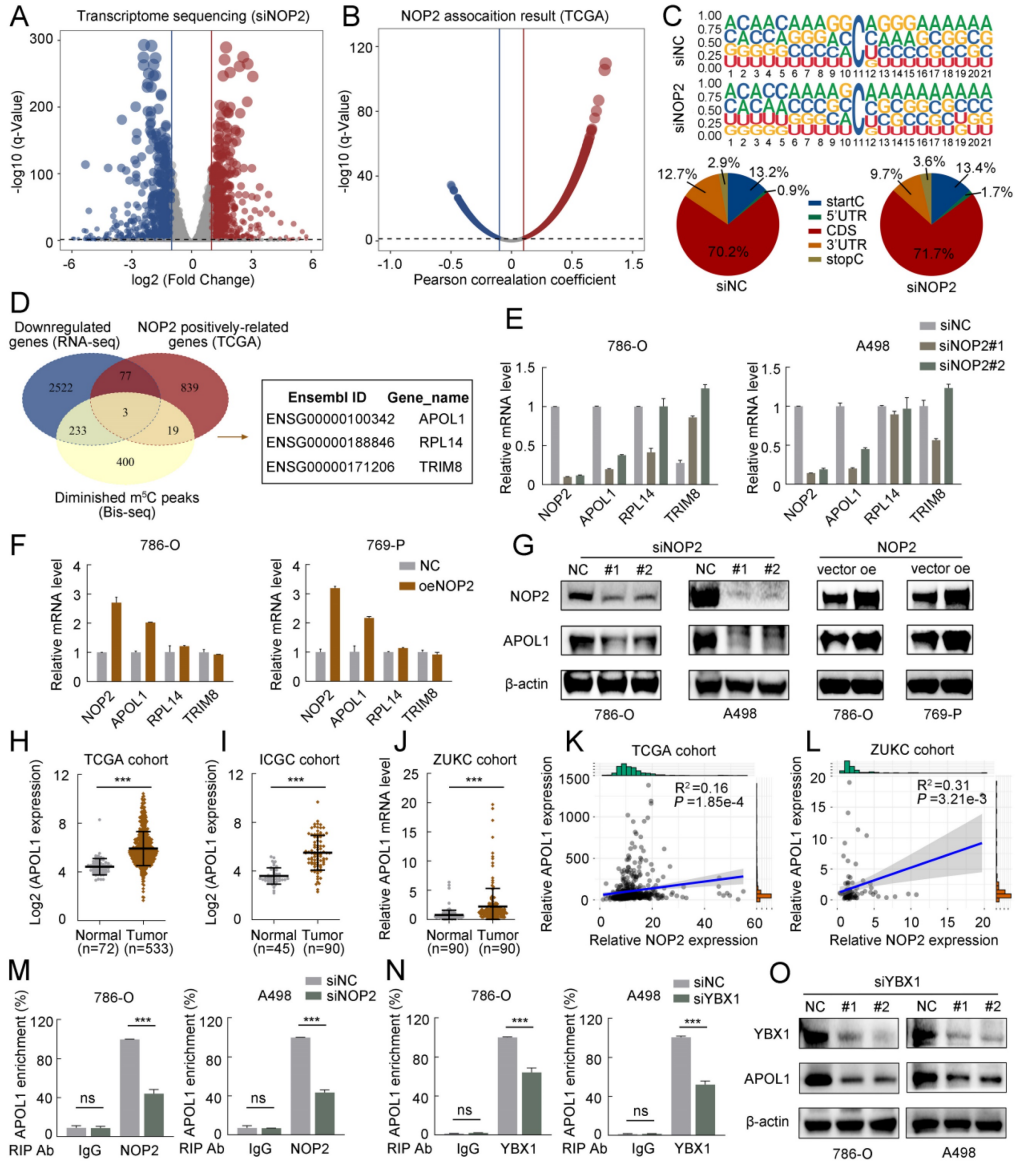
** A high-throughput sequencing combination revealed APOL1 to be the potential target of NOP2.** (**A**) Volcano plot showed changes in differential genes after NOP2 knockdown. (**B**) Volcano plot showed the significant genes associated with NOP2 expression under the Pearson's correlation coefficient analysis. (**C**) m^5^C sequence frequency logo in GC transcripts and distribution of mRNA m^5^C sites in GC. (**D**) Venn plot displayed the intersected genes from RNA-seq (downregulated DEGs), Bis-seq, and NOP2 positively correlated genes. Three common genes were screened out. (**E, F**) The mRNA levels of overlapped genes in NOP2-knockdown (**E**) and NOP2-overexpressing (**F**) ccRCC cells were validated by RT-qPCR. (**G**) The protein level of APOL1 in NOP2-knockdown or NOP2-overexpressing ccRCC cells were detected by Western blotting. (**H**) The levels of APOL1 expression were analyzed in ccRCC (n=533) and peritumoral normal kidney tissues (n=72) using TCGA cohort. (**I**) The levels of APOL1 expression were analyzed in ccRCC (n=90) and peritumoral normal kidney tissues (n=45) using ICGC cohort. (**J**) The levels of APOL1 expression were detected in ccRCC and paired normal kidney tissues by RT-qPCR from ZUKC cohort (n=90). NOP2 expression was positively correlated with APOL1 expression in ccRCC from TCGA (**K**) and ZUKC cohort (**L**), respectively. RIP-qPCR detected the content of APOL1 mRNA immunoprecipitated by NOP2 (**M**) and YBX1 (**N**) specific antibodies. IgG antibodies were used as negative control. (**O**) APOL1 protein expression level was detected by Western blotting in 786-O and A498 cells upon knockdown of YBX1. Data were displayed as mean ± SD. Differences were considered significant at *P* < 0.05 (ns, non-significance, *** *P* < 0.001).

To further elucidate the underlying molecular mechanism by which NOP2 exerts tumor-promoting effects in ccRCC, we performed RNA sequencing (RNA-seq) and m^5^C bisulfite sequencing (Bis-seq) on 786-O cells with NOP2 knocked down (siNOP2) and control cells (siNC). Additionally, we analyzed the TCGA-KIRC cohort to identify the potential genes regulated by NOP2-mediated m^5^C modification. RNA-seq revealed significant changes in gene expression following NOP2 deletion: 749 upregulated differentially expressed genes (DEGs) and 937 downregulated DEGs were identified (with a fold change of |logFC| > 2 and an adjusted p < 0.05) (Fig. 4A). Additionally, in the TCGA-KIRC cohort, 2835 genes were positively correlated and 2794 were negatively correlated with NOP2 expression (with |Pearson| > 0.1 and adjusted p < 0.05) (Fig. 4B). Subsequently, we performed Bis-seq to map transcriptome-wide m^5^C modifications after NOP2 knockdown in 786-O cells. In total, we identified 5152 and 4845 m^5^C peaks in the control and NOP2-knockdown cells, respectively (Supplementary Fig. S4A). Our findings that m^5^C sites were localized in CG-rich environments were consistent with those previous reports 17, 18 (Fig. 4C). In addition, Bis-seq analysis categorized m5C peaks based on their locations within the gene structure: 5′-untranslated regions (5′-UTR), start codon segment (startC), coding sequence (CDS), stop codon segment (stopC), and 3′-untranslated regions (3′-UTR) (Fig. 4C). Intriguingly, three genes, including APOL1, RPL14, and TRIM8, overlapped in the RNA-seq data, TCGA transcriptome profiles, and Bis-seq data (Fig. 4D). Therefore, we verified the regulation of NOP2 in these genes in ccRCC cells using RT-qPCR. Among these, APOL1 was the most significantly down-regulated gene in ccRCC cells following NOP2 knockdown (Fig. 4E) and was upregulated when NOP2 was overexpressed, highlighting its potential role in ccRCC progression (Fig. 4F). Consistent with the mRNA expression, APOL1 protein expression levels of APOL1 displayed the same trend (Fig. 4G). Further analysis of APOL1 mRNA levels in the TCGA, ICGC, and ZUKC-ccRCC cohorts revealed that APOL1 was significantly upregulated in ccRCC tissues than in normal kidney tissues (Fig. 4H-J). We further investigated the correlation between APOL1 expression levels and clinicopathological features in ccRCC patients from the TCGA (Supplementary Table S3) and ZUKC cohorts (Supplementary Table S4). Survival analysis revealed that patients with high levels of APOL1 had poor OS, PFS, and DSS in the TCGA-KIRC cohort (Supplementary Fig. S3A-C). Moreover, univariate and multivariate Cox regression analyses showed that APOL1 expression levels served as an independent prognostic factor for OS in ccRCC patients (Supplementary Fig. S3D, E).

The m^5^C readers can recognize and bind to m^5^C-modified mRNAs, influencing protein expression and altering biological effects. Interestingly, YBX1, a known regulator of mRNA stability 31-33 was significantly overexpressed in ccRCC tissues than in normal kidney tissues (Supplementary Fig. S4B-D). Subsequently, we analyzed the correlation between APOL1 and NOP2/YBX1 expression in the TCGA and ZUKC cohorts. Consistent with our hypothesis, APOL1 expression significantly positively correlated with NOP2 (Fig. 4K, L) and YBX1 expression (Supplementary Fig. S4E, F). Moreover, RIP-qPCR experiments revealed that antibodies specific to NOP2 and YUXI considerably enriched APOL1 mRNA compared with that precipitated by IgG. Conversely, silencing NOP2 and YBX1 markedly decreased APOL1 mRNA enrichment (Fig. 4M, N). Furthermore, we observed a substantial reduction in YBX1-modified APOL1 following NOP2 knockdown (Supplementary Fig. S4G, H). Furthermore, YBX1 knockdown consistently downregulated the expression of APOL1 at both the mRNA level (Supplementary Fig. S4I) and protein level (Fig. 4O). However, no significant effect on APOL1 expression was observed at the mRNA or protein level after ALYREF knockdown (Supplementary Fig. S4J, K).

**Figure 5:**
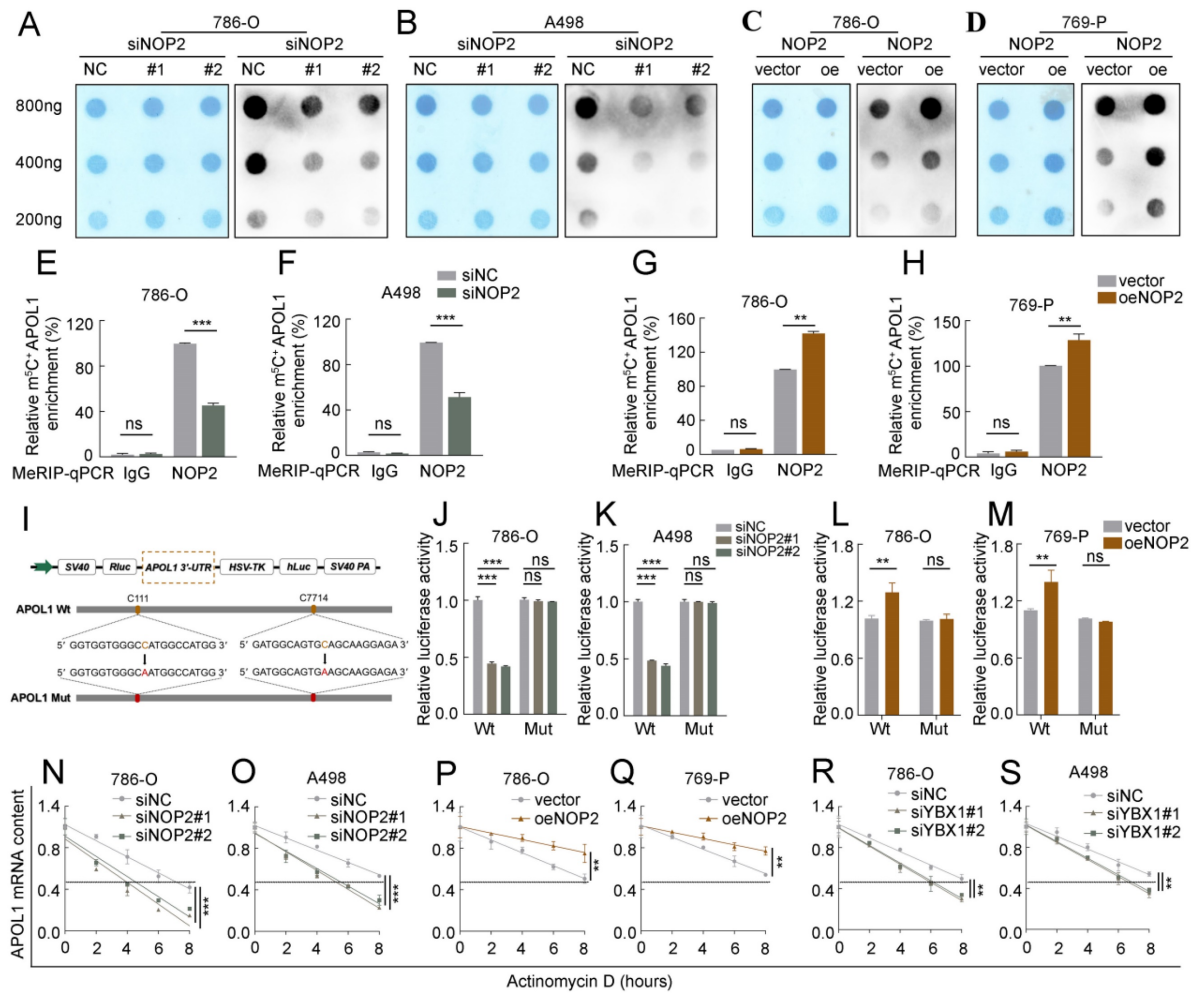
** NOP2-mediated m^5^C modification of APOL1 mRNA maintained its YBX1-dependent stability.** (**A-D**) The m^5^C level of total RNAs in ccRCC cells with knockdown (**A, B**) or overexpression (**C, D**) of NOP2. Methylene blue staining was used as a loading control. (**E-H**) MeRIP-qPCR analysis was performed to reveal NOP2-mediated APOL1 m^5^C modifications. The m^5^C modification of APOL1 was depleted on knockdown of NOP2 (**E, F**), while it was increased on up-regulation of NOP2 (**G, H**). (I) PsiCHECK2-APOL1-3′-UTR plasmid with either wild-type (Wt) or mutant (Mut) m^5^C sites were constructed based on Bis-seq data. (**J-M**) Relative luciferase activity of the Wt or Mut reporters in NOP2-depletion (**J, K**) and overexpression (**L, M**) ccRCC cells were detected (normalized to negative control groups). (**N-S**) The stability of APOL1 mRNA was determined in NOP2 knockdown (**N, O**), NOP2 overexpressing (**P, Q**), YBX1 knockdown (**R, S**) and their corresponding control ccRCC cells after treatment with Actinomycin D (5 µg/mL) at the indicated time points (normalized to 0 h).

Based on the Bis-seq data, we explored whether the tumor-promoting effects of NOP2 in ccRCC are dependent on its m^5^C catalytic activity. The dot-blot assay revealed that the total m^5^C levels were substantially reduced with the deletion of NOP2 at different RNA concentrations (Fig. 5A, B), whereas NOP2 overexpression increased total m^5^C levels (Fig. 5C, D). Next, we performed a MeRIP-qPCR assay to determine the enrichment of m^5^C in APOL1, which demonstrated that the m^5^C-specific antibody markedly enriched APOL1 transcripts compared with that in IgG controls. However, following NOP2 depletion, m5C-modified APOL1 decreased notably (Fig. 5E, F). Conversely, NOP2 overexpression increased the m^5^C levels in APOL1 mRNA (Fig. 5G and H). To further substantiate the role of m^5^C modification in APOL1 mRNA regulated by NOP2, we constructed wild-type (Wt) and mutant (Mut) 3′-UTR of APOL1 reporter plasmids for luciferase reporter assays. In the mutant APOL1 plasmids, the m^5^C consensus sequences were altered by changing cytosine (C) to adenine (A) at positions C111 and C7714, based on Bis-seq data. (Fig. 5I). As anticipated, NOP2 depletion substantially reduced the relative luciferase activity of Wt 3′-UTR of the APOL1 reporter gene; however, the decreased luciferase activity was partially abrogated in the case of mutated m^5^C sites (Fig. 5J, K), suggesting that NOP2 regulates APOL1 through m^5^C modification. Additionally, these results were verified in NOP2-overexpressing Wt and Mut plasmids (Fig. 5L, M). Considering that m^5^C modification positively regulates APOL1 mRNA levels, we evaluated whether m^5^C modification affects the stability of APOL1 mRNA. We found that knockdown or overexpression of NOP2 shortened or prolonged the half-life of APOL1 mRNA under the influence of actinomycin D (Fig. 5N-Q). Additionally, APOL1 mRNA stability was moderately decreased by YBX1 knockdown (Fig. 5R, S). Taken together, these results revealed that the methylated APOL1 transcripts were directly recognized by the m^5^C “reader” YBX1, which maintained the stability of the transcripts to prevent their degradation and naturally increase their expression through an m^5^C-YBX1-dependent mechanism.

### APOL1 was involved in NOP2-mediated ccRCC malignant process *in vitro* and *in vivo*

**Figure 6:**
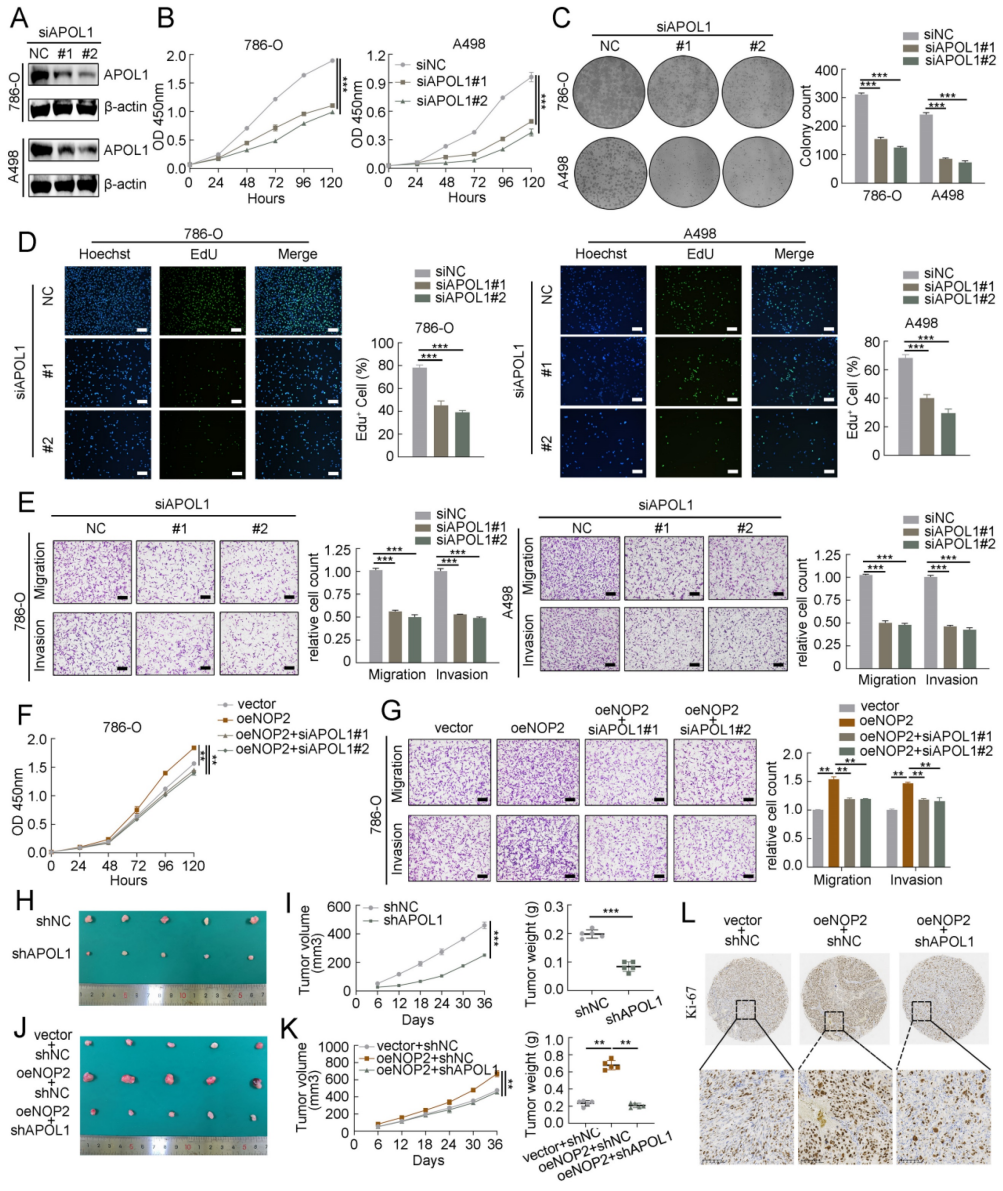
** APOL1 was involved in NOP2-mediated ccRCC malignant process *in vitro* and* in vivo*.** Western blotting analysis of APOL1 knockdown efficiency in 786-O and A498 cells. (**B-D**) The proliferation of ccRCC cells under silenced APOL1 was detected via CCK-8 (**B**), colony-formation (**C**), and EdU assays (**D**). (**E**) Knockdown of APOL1 impaired cell migration and invasion ability in 786-O and A498 cells, with bar charts indicating the quantification results of cell migration and invasion (right panel). Rescue experiments were conducted to determine the influence of down-regulated APOL1 with overexpressing of NOP2 in cells proliferation (**F**) and cells migration and invasion abilities (**G**). (**H**) Tumor growth nodules of stable APOL1 knockdown and negative control 786-O cells in the xenograft mouse model were shown, (**I**) followed by the generation of tumor growth curve and tumor weight records. (**J**) Knockdown of APOL1 inhibited NOP2-induced 786-O cells subcutaneous tumour growth in nude mice (n=5). (**K**) The tumor growth curve and tumor weight records were shown after 5 weeks. (**L**) Sections of nude mice subcutaneous tumors were stained with anti-Ki67 antibodies by IHC. Scale bar, 50 μm. Data were displayed as mean ± SD. Differences were considered significant at *P* < 0.05 (** *P* < 0.01, *** *P* < 0.001).

To further investigate the oncogenic function of APOL1 in ccRCC, two different siRNAs targeting APOL1 were used, and the knockdown efficiency was confirmed by western blotting (Fig. 6A). APOL1 knockdown significantly suppressed cell proliferation rate, clonogenic ability (Fig. 6B-D), cell migration, and invasion (Fig. 6E; Supplementary Fig. S4A), and induced apoptosis (Supplementary Fig. S4B, C). Additionally, to explore whether NOP2 accelerates ccRCC malignancy by regulating APOL1 expression. As expected, the expression of APOL1 was knocked down in NOP2-overexpressed 786-O and 769-P cells using specific siRNAs, which moderately suppressed NOP2-induced ccRCC cell proliferation (Fig. 6F; Supplementary Fig. S4D), colony formation (Supplementary Fig. S4E), migration, and invasion abilities (Fig. 6G; Supplementary Fig. S4F). In addition, we used a subcutaneous ccRCC mouse model to determine the role of APOL1 *in vivo*, which indicated that APOL1 silencing markedly suppressed tumor growth in the mice model than in the controls (Fig. 6H, I). Moreover, NOP2 overexpression increased tumor size and weight in subcutaneous ccRCC mice; however, these effects were mitigated by APOL1 knockdown (Fig. 6J-L).

### Elevated NOP2 induced PI3K-Akt signaling activation through the regulation of APOL1

**Figure 7:**
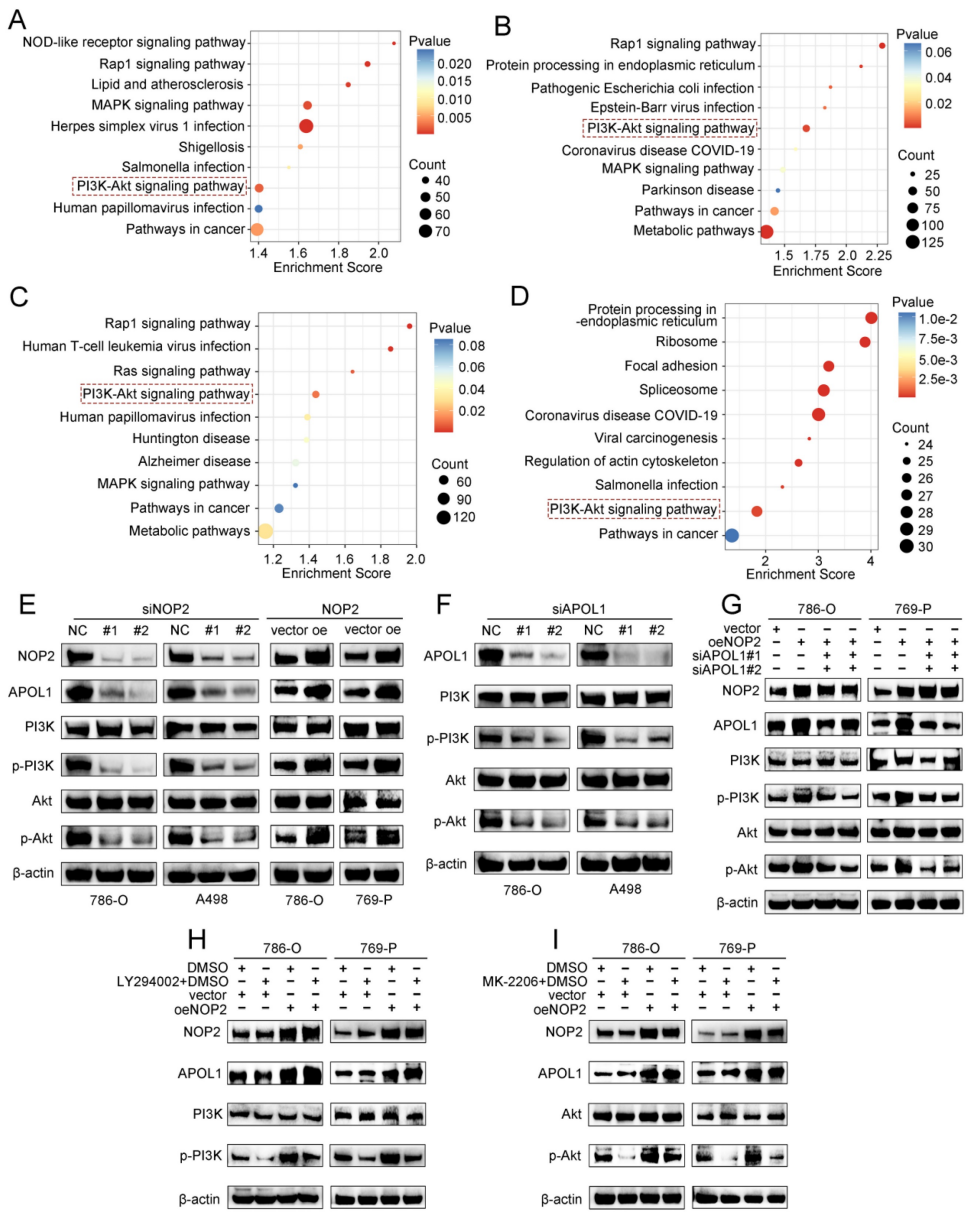
** NOP2/APOL1 was involved the progression of ccRCC via PI3K-Akt pathway.** (A)KEGG pathway analysis of NOP2-regulated genes in NOP2-deficient 786-O cells. (**B**) KEGG pathway analysis of differentially expressed genes between the APOL1^high^ and APOL1^low^ group in TCGA-KIRC cohort. (**C**) KEGG pathway analysis of differentially expressed genes between the NOP2^high^ and NOP2^low^ group in TCGA-KIRC cohort. (**D**) KEGG pathway analysis of m^5^C-modified genes in NOP2-deficient 786-O cells. (**E**) The phospho-PI3K and phospho-Akt protein expression in ccRCC cells with knockdown or overexpression of NOP2. (**F**) The phospho-PI3K and phospho-Akt protein expression in ccRCC cells with depletion of APOL1. (**G**) The phospho-PI3K and phospho-Akt protein expression levels in down-regulation of APOL1 with NOP2-overexpressing ccRCC cells. Overexpression of NOP2 could significantly rescued the reduced phospho-PI3K and phospho-Akt protein expression level by PI3K (**H**) and Akt (**I**) inhibitor, respectively. LY294002: PI3K inhibitor; MK2206: Akt inhibitor.

To further elucidate the underlying molecular mechanism by which APOL1 is involved in NOP2-mediated ccRCC malignant process, we conducted pathway enrichment analysis of DEGs using the Kyoto Encyclopedia of Genes and Genomes (KEGG) database. We analyzed DEGs from RNA-seq after NOP2 knockdown (Fig. 7A), DEGs between the APOL1^high^ and APOL1^low^ groups (Fig. 7B), DEGs between the NOP2^high^ and NOP2^low^ groups (Fig. 7C), and NOP2-mediated m^5^C-modified DEGs (Fig. 7D). The analysis revealed significant enrichment in several cancer-related signaling pathways, such as the metabolic, PI3K-Akt, MAPK, and Rap1 signaling pathways. Interestingly, we found that the PI3K-Akt signaling pathway was the only overlapping potential candidate pathway. As previously reported, the PI3K-Akt signaling pathway regulates the malignant behavior of various cancers, including ccRCC 34-38. Therefore, we hypothesized that NOP2/APOL1 are involved in ccRCC progression by regulating the PI3K-Akt signaling pathway. Western blotting showed that the phosphorylation levels of PI3K (p85) and Akt (Ser473) were significantly suppressed in cells with NOP2 knockdown, whereas NOP2 overexpression elevated the levels of phospho-PI3K and phospho-Akt (Fig. 7E). In addition, PI3K and Akt were dephosphorylated in 786-O and A498 cells after APOL1 silencing (Fig. 7F). Subsequently, APOL1 downregulation substantially alleviated the elevated NOP2-increased phospho-PI3K and phospho-Akt levels in both 786-O and 769-P cells (Fig. 7G). To further validate the indirect PI3K and Akt phosphorylation regulation of NOP2 via APOL1, PI3K (LY294002) and Akt (MK-2206) inhibitors were used to inhibit the PI3K/Akt pathway in ccRCC cells overexpressing NOP2. The results showed that NOP2 overexpression restored the levels of phospho-PI3K and phospho-Akt inhibited by LY294002 (Fig. 7H) and MK2206 (Fig. 7I), respectively. These results imply that NOP2/APOL1 mediates the malignant process in ccRCC via the PI3K-Akt pathway.

### Clinical significance of NOP2/APOL1 axis induced tumor progression in human ccRCC

To evaluate the clinical correlation between NOP2 and APOL1, immunohistochemical staining of NOP2 and APOL1 was performed using the ZUKC microarray (Supplementary Fig. S6A). Notably, NOP2 expression was positively correlated with APOL1 expression in ccRCC tissues (Supplementary Fig. S6B). As previously mentioned, high NOP2 or APOL1 expression was independently associated with poor prognosis of ccRCC patients in the TCGA cohort (Supplementary Fig. S1A-C; Supplementary Fig. S3A-C). Furthermore, Kaplan-Meier analysis revealed that, compared with other groups, ccRCC patients with high NOP2^high^ APOL1^high^ expression had worse OS (Supplementary Fig. S6C). These findings suggest that NOP2 and APOL1 co-expression are positively correlated in clinical samples and may serve as an effective prognostic indicator in ccRCC patients.

## Discussion

**Figure 8:**
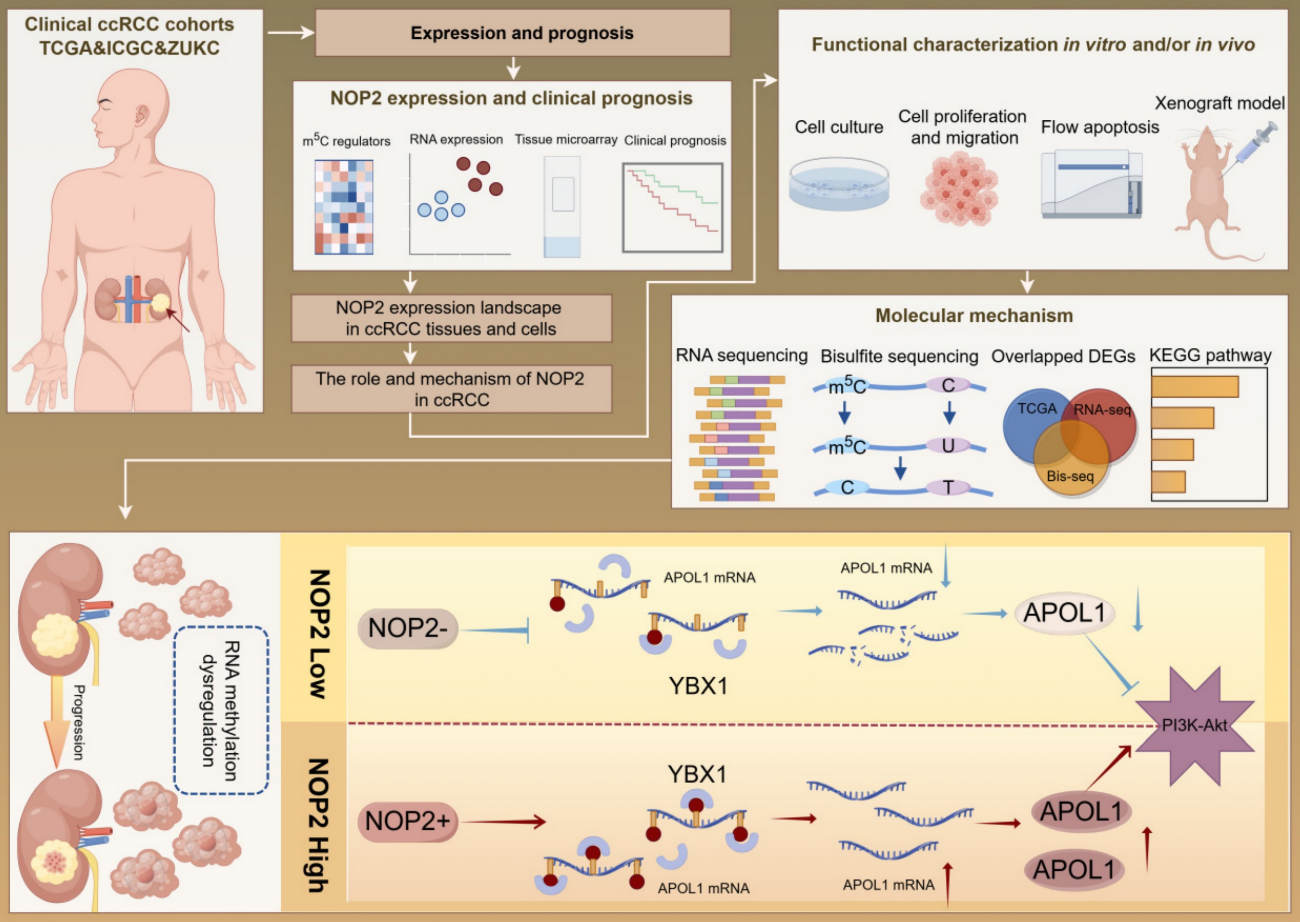
A graphical summary of methodology and regulatory mechanism.

Recently, RNA epigenetics/epitranscriptomics has received increased attention and interest 11-13. To date, over 100 types of chemical modifications of human RNA have been identified 14, 39. Among them, m^5^C, a critical RNA modification, has gained considerable attention because it dynamically regulates multiple physiological and pathological processes via its regulators in various organisms 40-42. The m^5^C modification is most abundant in tRNA, rRNA, and mRNA and affects RNA stability 43, translational fidelity 44, cell differentiation 45, nervous system regulation, reproductive system development 46, 47, and viral viability 48. Recent advances in detection methods have enabled extensive investigation of mRNA m^5^C modifications. Abnormal mRNA m^5^C modifications are associated with the development and progression of multiple diseases 19, 49. Our findings indicated that NOP2 was significantly upregulated in ccRCC cells and tissues and was associated with poor prognosis in patients with ccRCC. Functionally, altering NOP2 expression through loss-of-function and gain-of-function assays altered the growth and progression of ccRCC. Mechanistically, NOP2 facilitated m^5^C modification of APOL1 mRNA, and m^5^C reader YBX1 stabilized APOL1 mRNA through recognizing and binding to the m^5^C site in 3′-untranslated regions. Finally, NOP2 promoted ccRCC progression by maintaining APOL1 in an m^5^C-dependent manner, thereby activating the PI3K-Akt signaling pathway (Fig. 8).

As a member of the m^5^C methyltransferase family, elevated NOP2 promotes cell progression in colon cancer 20 and hepatocellular carcinoma 21 and is associated with poor prognosis in most cancers 50. However, NOP2 has rarely been investigated in terms of tumor progression related to its m^5^C modification activity, particularly in ccRCC. Consistent with the results of previous studies, we found that expression of m^5^C regulators was dysregulated in ccRCC. NOP2 expression was closely correlated with other m^5^C regulators and was most significantly associated with prognosis in ccRCC patients. Subsequent studies confirmed that NOP2 induces an increase in m^5^C modifications in ccRCC cells. Further prognostic analysis showed that elevated NOP2 expression was associated with poor prognosis in ccRCC patients and that incorporating NOP2 expression could assist in the predictive ability of the clinical prognostic model, suggesting that NOP2 may be a biomarker for ccRCC prognosis. Furthermore, *in vitro* and* in vivo* functional studies have demonstrated that NOP2 promotes ccRCC progression depending on its m^5^C catalytic activity. Therefore, NOP2 may serve as a potential prognostic biomarker and therapeutic target in ccRCC patients. To further elucidate the underlying molecular mechanism of NOP2 by combining the data from RNA-seq, Bis-seq, and TCGA transcriptomic profiles, we showed that APOL1 is a potential downstream target of NOP2. Subsequently, MeRIP-qPCR, luciferase reporter, and RNA decay assay results indicated that APOL1 was positively regulated by NOP2 and was modified in the 3′-UTR through NOP2-dependent m^5^C modification.

APOL1 acts as a minor component of secreted high-density lipoprotein, which binds to apolipoprotein A1 and participates in lipid transport and metabolism 51-53. Dysregulated APOL1 is involved in multiple biological processes in various diseases 54-57. The expression of APOL1 was prevalently upregulated in several cancers, including head and neck squamous cell carcinoma 58, papillary thyroid carcinoma 59, small cell lung carcinoma 60, hepatocellular carcinoma 61, pancreatic cancer 57, and bladder cancer 62. In the current study, we demonstrated that APOL1 was significantly upregulated and related to poor prognosis in ccRCC patients. Subsequently, we functionally confirmed that APOL1 silencing impaired the proliferation, migration, and invasion of ccRCC cells. In addition, silencing APOL1 partly rescued the overexpression of NOP2-induced promotion of ccRCC progression, which highlighted the vital role of APOL1 in NOP2-driven ccRCC progression. Finally, we assessed the downstream pathways underlying NOP2/APOL1 in promoting ccRCC progression. The PI3K-Akt pathway is involved in the progression of various cancers through m^5^C modification 35-37. Notably, the preliminary results of the KEGG pathway enrichment analysis showed that NOP2/APOL1 may be involved in the regulation of PI3K-Akt pathway. Furthermore, western blotting revealed that the PI3K-Akt pathway was markedly repressed by silencing NOP2/APOL1. Subsequently, APOL1 silencing alleviated NOP2-increased PI3K-Akt phosphorylation. Furthermore, NOP2 overexpression rescued the low PI3K/Akt phosphorylation levels inhibited by PI3K/Akt pathway inhibitors. Therefore, these results indicate that elevated NOP2 activates PI3K-Akt signaling through the regulation of APOL1 to promote the progression of ccRCC.

However, our study had certain limitations. To investigate the potential downstream targets and specific molecular mechanisms of NOP2 regulation, we conducted a cross-analysis using high-throughput sequencing combined with TCGA transcriptome profiles. However, the final screened transcripts enriched for m^5^C modification may not have been sufficient, and m^5^C-associated candidates specifically involved in ccRCC may have been missed, warranting further investigation. Therefore, performing additional CLIP-seq and RIP-seq analyses could provide more comprehensive target identification. Additionally, the prevalence of m^5^C modification of APOL1 in different types of cancer cells requires further validation. In addition, *in vivo* studies using patient-derived tumor xenografts and tail vein injection models should be conducted to confirm our hypothesis. Finally, the possibility that NOP2 has an unfavorable role beyond m^5^C-related mechanisms in ccRCC progression deserves further investigation.

In summary, we identified NOP2 expression as an independent prognostic factor that correlated with poor clinical outcomes in ccRCC patients. Mechanistically, NOP2 stimulated m^5^C modification of APOL1 mRNA, and the m^5^C reader YBX1 stabilized APOL1 mRNA through recognizing and binding to the m^5^C site in the 3′-untranslated regions and subsequently promoted ccRCC progression via the PI3K-Akt signaling pathway. Therefore, NOP2/APOL1 may be a potential prognostic predictor and therapeutic target for ccRCC.

## Materials and methods

### Samples and databases

We acquired transcriptional and clinical data of KIRC cohort from The Cancer Genome Atlas (TCGA) database (https://portal.gdc.cancer.gov/), which included 533 KIRC samples and 72 paired paracancerous kidney tissues. Fragments per kilobase million values were used to compare DEGs among KIRC samples. RNA-seq data from the Renal Cell Cancer-European cohort, which comprised 90 primary tumor tissues and 45 non-tumor specimens were downloaded from the ICGC Data Portal (https://dcc.icgc.org/). In addition, we randomly collected tumor and paired paracancerous kidney tissues from 90 ccRCC patients who had undergone radical or partial nephrectomies. All samples were obtained from the First Affiliated Hospital, Zhejiang University School of Medicine, between January 2020 and December 2023. This study was conducted in accordance with the principles of medical ethics and was approved by the Institutional Ethics Committee of the First Affiliated Hospital, Zhejiang University School of Medicine.

### Cell culture

Human normal renal epithelial cell line (HK-2) and renal cell carcinoma cell lines (786-O, 769-P, ACHN, Caki-1, and A498) were purchased from the National Collection of Authenticated Cell Cultures (Shanghai, China) and authenticated by STR. HK-2, 786-O, and 769-P cells were cultured in Roswell Park Memorial Institute (RPMI) 1640 medium (Gibco, New York, USA) containing 10% fetal bovine serum (FBS) (ExCell Bio, Shanghai, China). A498, ACHN, and Caki-1 cells were maintained in Dulbecco's modified Eagle's medium (Gibco, New York, USA) with 10% FBS. All cells were cultured at 37 °C with 5% CO2.

### RNA interference

Small interfering RNA (siRNA) oligonucleotides against NOP2, APOL1, YBX1, ALYREF, and negative control RNAs were synthesized by SUNYA (Hangzhou, China; Supplementary Table S5). Cell transient transfection was performed using the jetPRIME^®^ Transfection Reagent (Polyplus, France) according to the manufacturer's instructions. Briefly, cells were plated in a 6-well or 6-cm plates (NEST, Wuxi, China), grown to 30-50% confluency (adherent cells), and then transfected and cultured at 37 °C for an additional 48 h, followed by RT-qPCR, western blotting, or other functional assays.

### Construction of stable knockdown and overexpressed cells

Plasmids for NOP2 knockdown, APOL1 knockdown, NOP2 overexpression, and the control (green fluorescent protein) were synthesized by GeneChem (Shanghai, China). The target plasmids were then co-transfected with packing and PAX2 plasmids into HEK 293T cells according to the manufacturer's instructions. After 48 h or 72 h of transfection, the lentivirus was collected for infecting 786-O, A498 and 769-P cells for 48 h. Subsequently, puromycin (2 μg/mL, MCE, Austin, USA) was applied for screening stably transfected target cells for 1-2 weeks. All target sequences are listed in Supplementary Table S5.

### RNA extraction and RT-qPCR

Total RNA was isolated from cultured cells and tissue samples using Trizol reagent (Life Technologies, Ambion^®^, Austin, Texas, USA), followed by cDNA synthesis with HiScript^®^ II Q RT SuperMix for qPCR (Vazyme Biotech, Nanjing, China). ChamQ Universal SYBR qPCR Master Mix (Vazyme Biotech, Nanjing, China) with a PCR detection system (Bio-Rad, CFX96TM, Hercules, CA, USA) was used to measure RNA expression. The relative target gene expression level was detected by the 2^-ΔΔCt^ calculation method with normalization to GAPDH or β-actin. All primers were obtained from Tsingke Biological Technology (Beijing, China) and are listed in Supplementary Table S5.

### Western blotting

The cells were harvested and lysed in radioimmunoprecipitation assay (RIPA) lysis buffer in the presence of protease and phosphatase inhibitors (Beyotime, Shanghai, China). The protein concentration was determined using a bicinchoninic acid protein assay kit (Beyotime, Shanghai, China). Whole cell lysates were subjected to sodium dodecyl sulfate-polyacrylamide gel electrophoresis and transferred to polyvinylidene fluoride membranes (Immobilon P; Millipore, Burlington, MA, USA). After blocking with 5% bovine serum albumin (BSA) and incubating with specific primary and secondary antibodies, the proteins were visualized using the Bio-Rad ChemiDoc^®^ Touch Imaging System (Bio-Rad, CA, USA). All the antibodies used in this study are listed in Supplementary Table S6.

### Immunohistochemistry (IHC)

IHC staining of tissue microarray-based paraffin-embedded ccRCC samples from the ZUKC cohort was performed to evaluate the expression of target proteins. IHC staining was performed using a two-step Dako Envision™ Detection System (DakoCytomation, Glostrup, Denmark). Briefly, sections were incubated with target-specific primary antibodies, followed by incubation with horseradish peroxidase (HRP)-conjugated secondary antibodies, counterstained with hematoxylin, and visualized using an inverted microscope (Olympus, Tokyo, Japan). All antibodies used are listed in Supplementary Table S6. The IHC score was calculated by multiplying the staining intensity score with the positive rate score. Staining intensity scores of 0, 1, 2, and 3 represented negative, light, moderate, and strong staining, respectively. According to the percentage of positive cells, scores of 0, 1, 2, 3, and 4 indicated positive areas of 0%, < 10%, 10%-50%, 50%-80%, and > 80% positive cells, respectively. The final score ranged from 0 to 12, and scores of 0-6 and 7-12 were determined as low and high expressions, respectively. Two proficient pathologists independently calculated the scores.

### Cell proliferation assays

Cell proliferation capability was assessed using Cell Counting Kit-8 (CCK-8), 5-ethynyl-2′-deoxyuridine (EdU), and colony formation assays as previously described 63. For the CCK-8 assay, 3 × 10^3^ ccRCC cells were seeded into each well of a 96-well plate and cultured at 37 °C and 5% CO2 in a cell incubator. CCK-8 reagent (MCE^®^ MedChem Express, Monmouth Junction, NJ, USA) diluted in the culture medium was added to the wells at 0, 24, 48, 72, 96 and 120 h. Absorbance values at 450 nm were detected using a microplate reader (BioTek, Synergy Neo2, Winooski, VT, USA). For the EdU assay, the cells were seeded in 96-well plates at a density of 1 × 10^4^ cells/well (three replicates), and 100 µL of medium containing 50 µM EdU (UElandy, Suzhou, China) was added and incubated at 37 °C for 2 h. Subsequently, the cells were fixed with 4% paraformaldehyde for 20 min and permeabilized with 0.1% Triton X-100 for 10 min. Finally, cells were stained with EdU YF® 488 Azide for 30 min, followed by the staining of nuclei with Hoechst 33342 for 30 min. The proportion of EdU-positive cells was visualized using an inverted fluorescence microscope (Olympus, Tokyo, Japan). For the colony formation assay, 2 × 10^3^ cells were cultured in each well of 6-well plates and cultivated for 1-2 weeks. Cells were stained with 0.1% crystal violet and fixed with 4% paraformaldehyde.

### Migration and invasion assays

Cell migration was investigated using wound healing and migration experiments. For the wound healing assay, the transfected ccRCC cells were grown to 90% confluency in 6-well plates, followed by the creation of a straight artificial wound using a 200-μL pipette tip in each well. To ensure consistent observation positions and reproducible cell migration results, images of the wounds were captured at the positive middle position of each 6-well plate at time zero and at 48 hours, and three technical replicates and three biological replicates were performed independently in separate 6-well plates. The migration assay was performed in an 8-mm transwell chamber with an 8-μm pore size filter (Corning, Washington, DC, USA). A total of 3 × 10^4^ transfected ccRCC cells with serum-free medium were inoculated in the upper chamber, and 600 μL of medium containing 10% FBS was added to the lower chamber. After incubation at 37 °C for 48 h, cells on the underside of the membrane were immobilized with 4% paraformaldehyde and stained with crystal violet. Similarly, invasion assay was completed using a transwell chamber with an 8-μm filter insert (Corning, Washington, DC, USA) with pre-coated diluted Matrigel (BD Biosciences). The penetrated cells were counted, and cell invasion was quantified using an inverted microscope (Olympus, Tokyo, Japan).

### Flow cytometry analysis

The transfected ccRCC cells were harvested by trypsinization with 0.25% ethylenediaminetetraacetic acid and transferred to polystyrene FACS tubes for double staining with annexin V-FITC/propidium iodide (PI), according to the manufacturer's protocol (Annexin V-FITC/PI Apoptosis Kit, MultiSciences, Hangzhou, China). The percentage of stained cells was analyzed using flow cytometry (CtytoFLEX, Beckman, USA) and FlowJo software version 10.6.2 (FlowJo, LLC, Ashland, USA). Among them, single-color positive control cells treated with an apoptosis-positive control solution were used to adjust compensation, which requires further investigation. However, the observed differences were more reasonable using a positive compensating control for PI with heat shock-induced apoptosis. Therefore, to enhance the reliability of the results, future studies should consider incorporating both drug and heat-shock-induced apoptosis as positive controls to cross-validate the approach.

### Xenograft model in nude mice

Approximately 1 × 10^6^ stably transfected 786-O cells were inoculated subcutaneously into the axillae of BALB/c nude mice (aged 4-5 weeks, female, five mice per group). The width (W) and length (L) of the subcutaneous tumors were measured weekly, and tumor volume (V) was estimated as follows: V = (W^2^ × L/2). At the end of the feeding period (4-6 weeks), the mice were euthanized. Subsequently, the subcutaneous tumors were isolated, weighed, and fixed in 4% formalin for IHC analysis. All animal studies were approved by the Institutional Animal Care and Use Committee of the First Affiliated Hospital First Affiliated Hospital, Zhejiang University School of Medicine (approval number: ST2023006).

### RNA-sequencing (RNA-seq)

Total RNA was isolated from NOP2 knockdown cells and their corresponding controls in 786-O cells. Library construction and RNA-seq were performed at Shanghai OE Biomedical Technology Co., Ltd. (Shanghai, China) using the Illumina HiSeq X Ten PE150 platform. The thresholds for screening DEGs were identified with |log2 (fold change)| > 2 and q-value < 0.05. Finally, DEGs were functionally annotated using KEGG pathway analysis.

### RNA Bis-seq and bioinformatics analyses

The m^5^C Bis-seq was conducted by CloudSeq Biotech Inc. (Shanghai, China). Briefly, rRNA-depleted RNA was bisulfite-converted and purified. Subsequently, RNA libraries were constructed, and sequencing was performed on an Illumina HiSeq 4000 instrument with 150-bp paired-end reads. After 3′ adaptor-trimming and removal of low-quality reads, the clean reads of Bis-treated libraries were aligned to the reference genome (UCSC HG19) using meRanGh software. Subsequently, meRanCall software was used to extract the methylated sites on the RNAs (peaks), and meRanCompare software was used to identify differentially methylated sites. Finally, the m5C methylated sites were annotated using Ensembl genome features, and the distribution of m^5^C methylated sites was plotted using the MetaPlot package (R software). In addition, KEGG pathway analysis was performed on the differentially methylated site-related genes.

### RIP assay

RIP assay was conducted using the Magna RIP™ kit (17-700, Millipore) in accordance with the manufacturer's instructions. Briefly, approximately 1 × 10^7^ cells were harvested and lysed in the RIP lysis buffer. Magnetic beads coated with 5 μg of specific antibodies against anti-NOP2 (Ab271075, Abcam), anti-YBX1 (Ab76149, Abcam) or control IgG were incubated with prepared cell lysates overnight at 4 °C. Subsequently, the beads containing immunoprecipitated RNA-protein complexes were incubated with proteinase K digestion buffer to remove proteins. The RNAs of interest were finally purified using phenol-chloroform RNA extraction methods and determined by RT-qPCR analysis, with normalization to the input.

### MeRIP assay

Total RNA was extracted using the TRIzol reagent. The MeRIP assay was conducted using the m^5^C MeRIP kit (GenSeq^®^, Shanghai, China) in accordance with the manufacturer's protocol. Briefly, total RNAs (˃ 100 μg) were chemically fragmented into approximately 200 nucleotides with 1× fragmentation buffer and allowed to precipitate overnight at -80 °C. Next, PGM magnetic beads coated with the anti-m^5^C antibody (Ab10805, Abcam) or control IgG were incubated with the fragmented RNAs in immunoprecipitation buffer for 1 h at 4 °C. Methylated RNA was then eluted and purified from the beads. Finally, the purified RNA and input controls were analyzed using RT-qPCR.

### RNA m^5^C dot blotting

Total RNA was extracted from NOP2 knockdown and overexpressing cells and their corresponding negative control cells, and the concentration was measured (Thermo Scientific, USA). The extracted mRNA samples were dissolved in three times the volume of RNA incubation buffer and denatured by heating at 65 °C for 5 min. Different amounts of RNA (200, 400, and 800 ng) were loaded onto Amersham Hybond N+ membranes (GE Healthcare, USA) fixed on a Bio-Dot apparatus (Bio-Rad, USA) in a mixture of ice-cold 20 × SSC buffer (Sigma-Aldrich, Germany). The membrane was cross-linked at 254 nm UV for 5 min on both the front and back sides after a short drying process. The membrane was then stained with 0.02% methylene blue in 0.3 mol/L sodium acetate, followed by scanning to ensure the consistency of the total input RNA content. After blocking with 5% BSA and incubating with an anti-m^5^C antibody (ab10805, Abcam, USA) and the corresponding HRP-conjugated anti-mouse secondary antibody, the intensity of the dot blot was visualized using an imaging system (Bio-Rad, USA).

### Luciferase reporter assays

Based on Bis-seq data, Wt and Mut 3′-UTR of the APOL1 reporter plasmid were constructed by Quanyang Biotechnology (Shanghai, China). The Wt and Mut plasmids were transfected into ccRCC cells, and the Dual-Luciferase Report Assay Kit (Vazyme Biotech, Nanjing, China) was used to detect luciferase activity according to the manufacturer's instructions. Each experiment was performed in triplicate.

### RNA stability assay

To evaluate the RNA stability, knockdown of NOP2 or YBX1 cells and corresponding Wt cells were exposed to actinomycin D (5 μg/mL, Sigma-Aldrich). The cells were collected at different time points (0, 2, 4, 6, and 8 h). Total RNA was extracted and subjected to RT-qPCR to assess the relative levels of APOL1 mRNA (0 h as a reference).

### Statistical analyses

All statistical analyses were performed using GraphPad Prism 9.0 (La Jolla, CA, USA) and R v4.3.1 (https://www.r-project.org/). Quantification data were presented as the mean ± standard deviation. One-way analysis of variance or two-tailed Student's t-test was conducted to compare continuous variables, and the non-parametric chi-square test was used to assess categorical variables. The Cox proportional hazards regression model was used for univariate and multivariate analyses. Kaplan-Meier curves and log-rank tests for significance were used for survival analysis. Statistical correlations were calculated using Pearson's correlation coefficients. Differences were considered significant at a *P-*value < 0.05, with asterisks denoting the level of statistical significance (* *P* < 0.05, ** *P* < 0.01 and *** *P* < 0.001).

## Supplementary Material

Supplementary figures and tables.

## Abbreviations

APOL1: Apolipoprotein L1
Bis-seq: Bisulfite sequencing
CCK8: Cell counting kit-8
ccRCC: Clear cell renal cell carcinoma
CLIP-seq: Crosslinking Immunoprecipitation sequencing
DEGs: Differentially expressed genes
DSS: Disease-specific survival
EdU: 5-ethynyl-2′-deoxyuridine
FBS: Fetal bovine serum
ICGC: International Cancer Genome Consortium
IHC: Immunohistochemistry
KIRC: Kidney clear cell carcinoma
m^5^C: 5-methylcytosine
MeRIP-qPCR: methylated RNA immunoprecipitation-Quantitative real-time PCR
mRNA: Messenger RNA
Mut: Mutant
NOP2: NOP2/Sun RNA methyltransferase 1
OS: Overall survival
PFS: Progression-free survival
RECA-EU: Renal Cell Cancer-European
RIP-seq: RNA Immunoprecipitation sequencing
RNA-seq: RNA sequencing
RT-qPCR: Reverse-transcription quantitative polymerase chain reaction
siRNA: small interfering RNAs
TCGA: The Cancer Genome Atlas
TNM: Tumor Node Metastasis
UTR: Untranslated regions
Wt: Wild-type
ZUKC: Zhejiang University Kidney Clear Cell Carcinoma
