# Supplementary material for: NOP2-mediated 5-methylcytosine modification of APOL1 messenger RNA activates PI3K-Akt and facilitates clear cell renal cell carcinoma progression
Source: Int J Biol Sci. 2024 Sep 9;20(12):4853–71. doi: 10.7150/ijbs.97503 (PMC11414376; doi:10.7150/ijbs.97503)
Supplement: Supplementary file 1 — Supplementary figures and tables. [file ijbsv20p4853s1.pdf]

Supplementary materials：

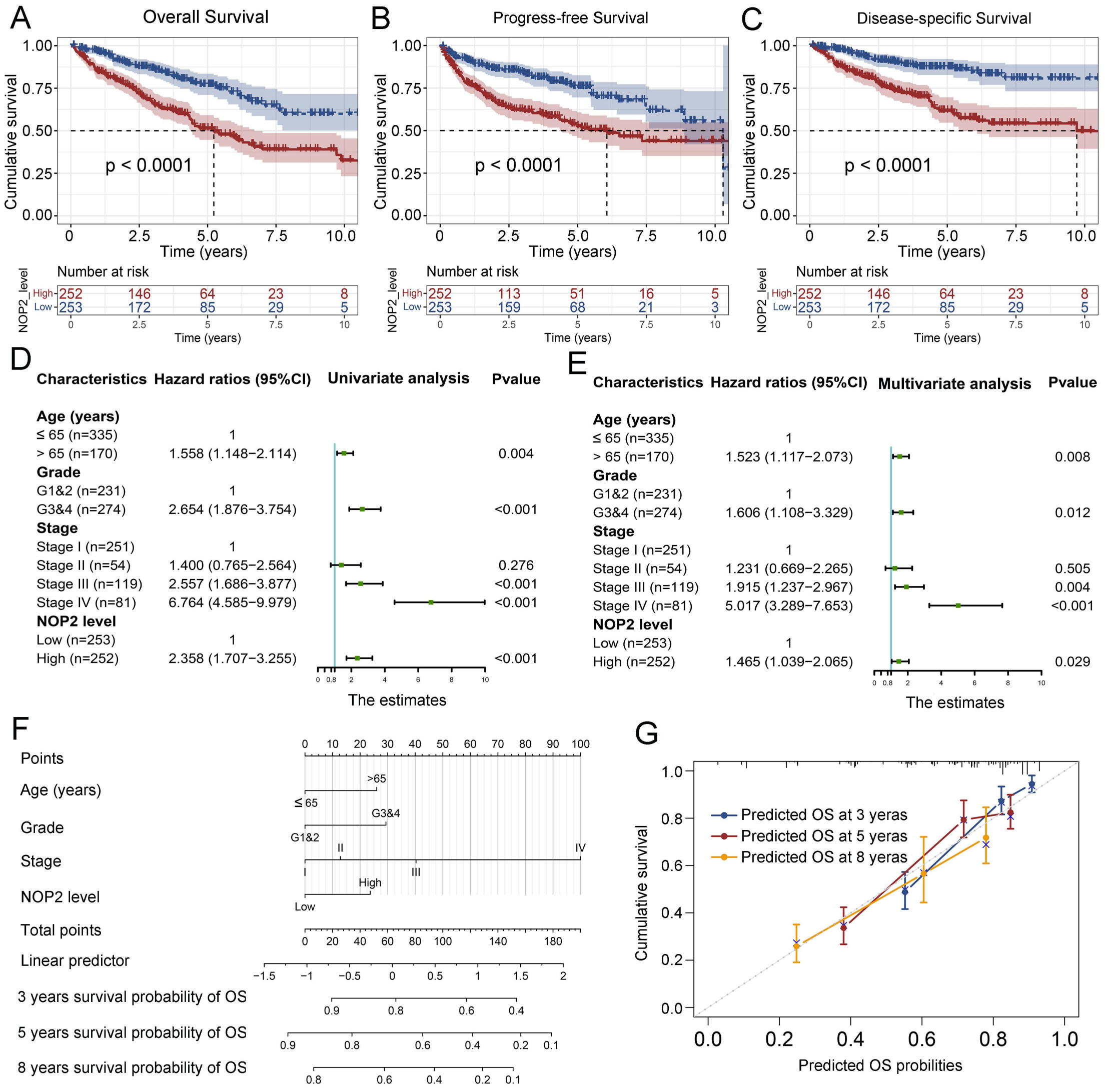

Supplementary Fig. S1 Association between NOP2 expression level and clinical parameters of ccRCC patients and its prognostic significance.

(**A-C**) Kaplan-Meier analysis of overall survival (OS), progression-free survival (PFS), and disease-specific survival (DSS) of NOP2 expression level in ccRCC patients from TCGA database. Univariate (**D**) and multivariate (**E**) survival analyses for selecting prognostic factors. All bars corresponded to 95% CIs. Establishment (**F**) and evaluation (**G**) of the overall survival nomogram for ccRCC patients.

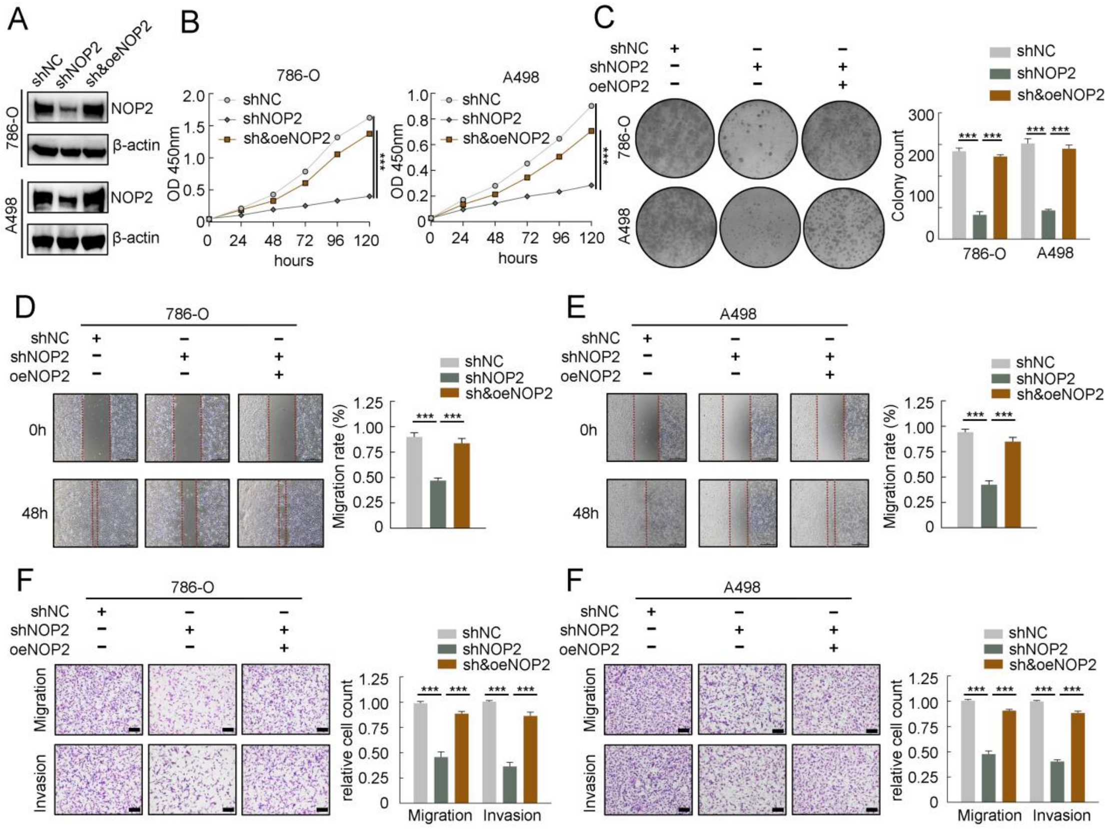

Supplementary Fig. S2 Re-expression of NOP2 in the knockdown cells completely rescued the defect in cell proliferation and migration ability.

(A) Western blotting analysis of NOP2 stable knockdown and re-expression (overexpression) efficiency in 786-O and A498 cells. (**B-C**) The proliferation of ccRCC cells under silenced and re-expressed NOP2 was detected via CCK-8 (**B**) and colony-formation (**C**) assays. Cell wound-healing assay (**D-E**), Transwell migration and invasion assay (**F-G**) revealed the effect of re-expression of NOP2 in the knockdown ccRCC cells, with bar charts indicating the quantification results of cell migration and invasion (right panel). Data were displayed as mean ± SD. Differences were considered significant at $P < 0.05$ (*** $P < 0.001$).

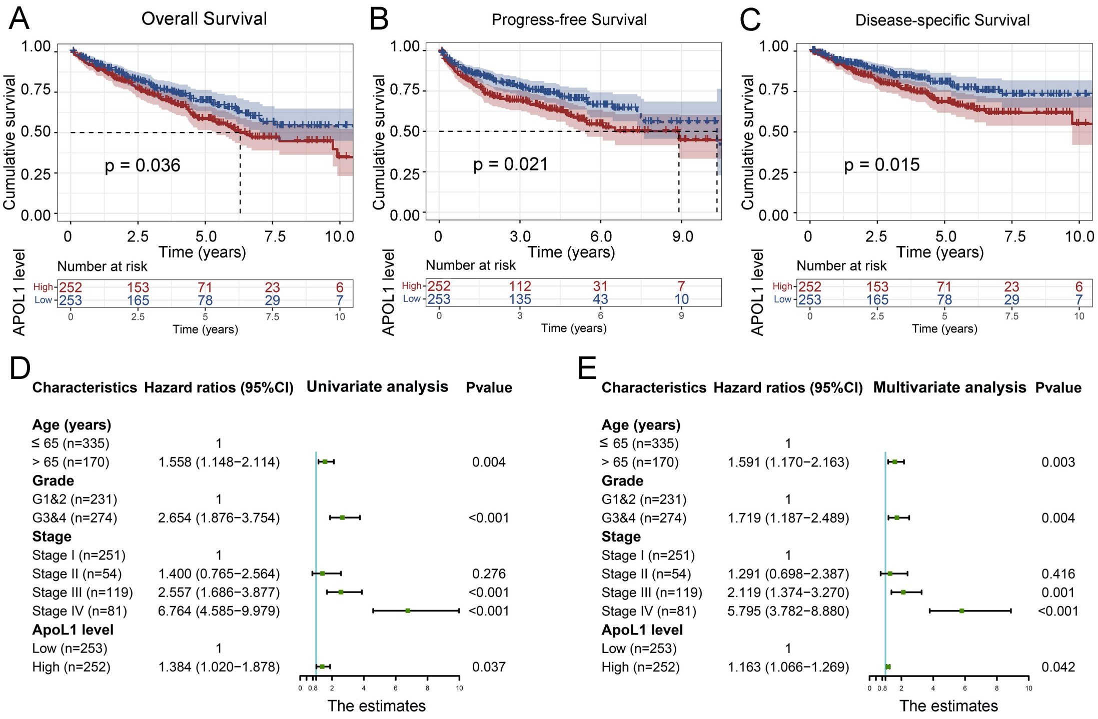

Supplementary Fig. S3 Up-regulated APOL1 expression was associated with poor outcomes of ccRCC.

(**A-C**) Kaplan-Meier analysis of overall survival (OS), progression-free survival (PFS), and disease-specific survival (DSS) of APOL1 expression level in ccRCC patients from TCGA database. (**D, E**) Univariate and multivariate survival analyses for selecting prognostic factors. All bars corresponded to 95% CIs.

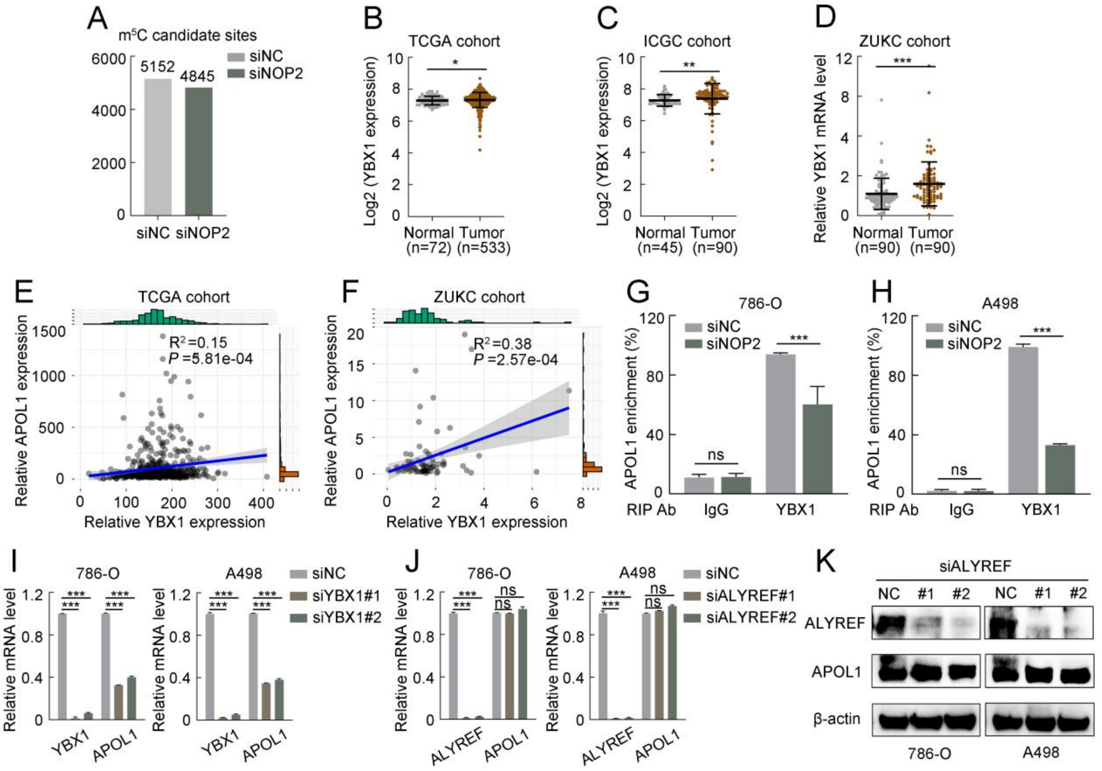

Supplementary Fig. S4 YBX1 serving as m^{5}C reader suppressed APOL1 expression.

(**A**) Bis-seq determined the m^{5}C peak numbers in control and NOP2-knockdown 786-O cells. (**B**) The level of YBX1 expression were analyzed in ccRCC (n=533) and peritumoral normal kidney tissues (n=72) using TCGA cohort. (**C**) The level of YBX1 expression were analyzed in ccRCC (n=90) and peritumoral normal kidney tissues (n=45) using ICGC cohort. (**D**) The level of YBX1 expression were detected in ccRCC and paired normal kidney tissues by RT-qPCR from ZUKC cohort (n=90). YBX1 expression was positively correlated with APOL1 expression in ccRCC from TCGA (**E**) and ZUKC cohort (**F**), respectively. RIP-qPCR detected the relative content of APOL1 mRNA immunoprecipitated by YBX1 specific antibodies in control (**G**) or NOP2-knockdown (**H**) cells. IgG antibodies were used as negative control. (**I**) APOL1 mRNA expression level was detected by RT-qPCR in 786-O and A498 cells upon knockdown of YBX1. (**J**) APOL1 mRNA expression level was detected by RT-qPCR in 786-O and A498 cells upon knockdown of ALYREF. (**K**) APOL1 protein expression level was detected by Western blotting in 786-O and A498 cells upon knockdown of ALYREF. Data were displayed as mean ± SD. Differences

were considered significant at $P < 0.05$ (ns, non-significance, * $P < 0.05$, ** $P < 0.01$, *** $P < 0.001$).

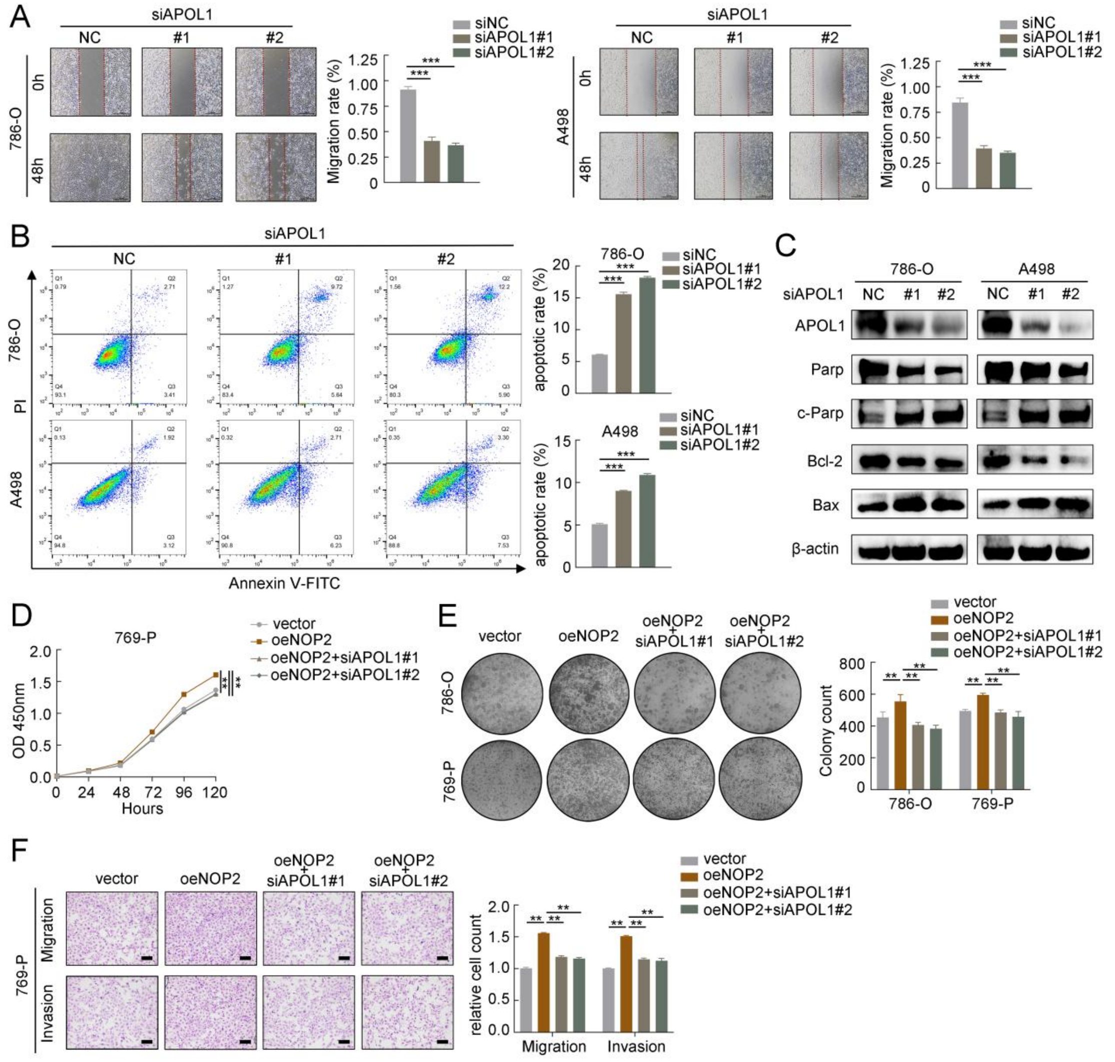

Supplementary Fig. S5 Down-regulated APOL1 attenuated the overexpression of NOP2-accelerated ccRCC malignant process.

(A) Cell wound-healing assay revealed the effect of APOL1 knockdown on 786-O and A498 cells. Knockdown APOL1 inducing apoptosis of ccRCC cells were detected by flow cytometry (**B**) and Western blotting assay (**C**). Rescue experiments were conducted to determine the influence of down-regulated APOL1 with overexpressing of NOP2 in cells proliferation (**D**), colony-formation (**E**), and cells migration and invasion abilities (**F**). The corresponding quantitative analysis results were presented in the right panel. Scale bar, 50 μm. Data were displayed as mean ± SD. Differences

were considered significant at *P* < 0.05 (** *P* < 0.01, *** *P* < 0.001).

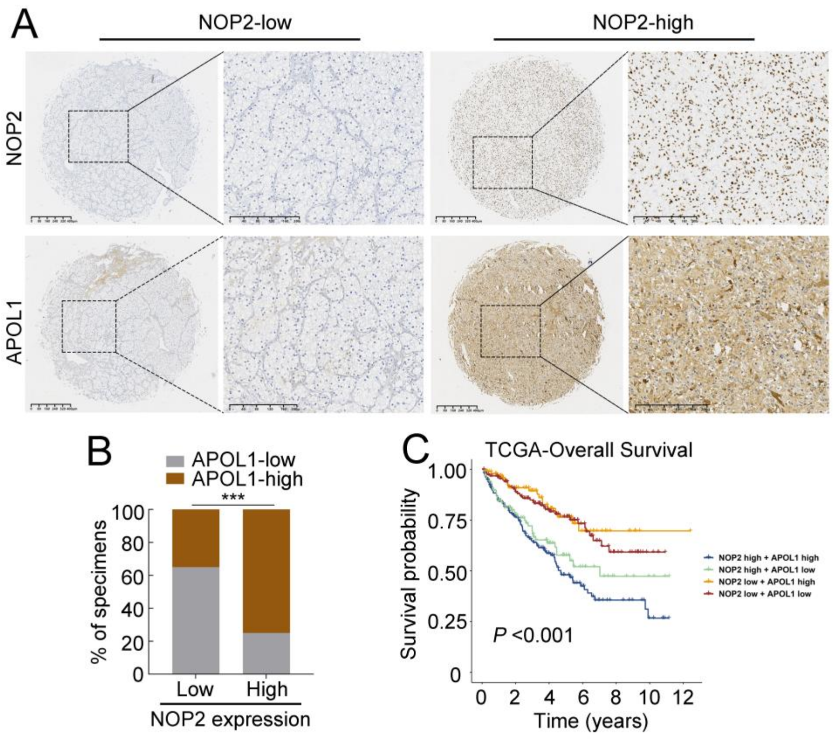

Supplementary Fig. S6 The clinical significance of NOP2/APOL1 axis induced tumor progression in human ccRCC.

(A) Representative IHC staining images showing high or low expression of NOP2 and APOL1 in the identical ccRCC tumor specimens from ZUKC cohort. (**B**) Correlation between NOP2 and APOL1 expression level in ccRCC microarray specimens. (**C**) Overall survival analysis based on the co-expression of NOP2 and APOL1 in ccRCC according to TCGA cohort. Differences were considered significant at *P* < 0.05 (*** *P* < 0.001).

Supplementary Table S1. Clinicopathological characteristics in relation to NOP2 expression level in the TCGA cohort.

Characteristics	Patients (n=505)	NOP2 expression		χ2	*P* value
		Low (%)	High (%)		
**Age**				1.58	0.209
<= 65	335	175 (52.2%)	160 (47.8%)		
> 65	170	78 (45.9%)	92 (54.1%)		
**Gender**				0.25	0.616
Male	333	170 (51.1%)	163 (48.9%)		
Female	172	83 (48.3%)	89 (51.7%)		
**Histologic grade**				44.37	***< 0.001***
Grade1	12	10 (83.3%)	2 (16.7%)		
Grade2	219	137 (62.6%)	82 (37.4%)		
Grade3	201	90 (44.8%)	111 (55.2%)		
Grade4	73	16 (21.9%)	57 (78.1%)		
**Pathologic stage**				51.81	***< 0.001***
Stage I	251	162 (64.5%)	89 (35.5%)		
Stage II	54	29 (53.7%)	25 (46.3%)		
Stage III	119	41 (34.5%)	78 (65.5%)		
Stage IV	81	21 (25.9%)	60 (74.1%)		
**T stage**				44.63	***< 0.001***
T1	257	163 (63.4%)	94 (36.6%)		
T2	65	33 (50.8%)	32 (49.2%)		
T3	172	54 (31.4%)	118 (68.6%)		
T4	11	3 (27.3%)	8 (72.7%)		
**N stage**				9.26	***0.008***
N0	226	121 (53.5%)	105 (46.5%)		
N1	15	2 (13.3%)	13 (86.7%)		
Nx	264	130 (49.2%)	134 (50.8%)		
**M stage**				29.24	***< 0.001***
M0	400	225 (56.3%)	175 (43.7%)		
M1	78	20 (25.6%)	58 (74.4%)		
Mx	27	8 (29.6%)	19 (70.4%)		
**OS event**				29.32	***< 0.001***
None	337	198 (58.8%)	139 (41.2%)		
Yes	168	55 (32.7%)	113 (67.3%)		
**PFS event**				19.69	***< 0.001***
None	346	197 (56.9%)	149 (43.1%)		
Yes	159	56 (35.2%)	103 (64.8%)		
**DSS event**				28.53	***< 0.001***
None	397	224 (56.4%)	173 (43.6%)		
Yes	108	29 (26.9%)	79 (73.1%)		

Statistical significance was determined by Chi-square test (if necessary, results were adjusted by Yate's correction) or Fisher's exact test (if n less than 5). Bold italics

indicate statistically significant values. TCGA: The Cancer Genome Atlas; OS: Overall survival; PFS: Progression free survival; DSS: Disease specific survival.

Supplementary Table S2. Clinicopathological characteristics in relation to NOP2 expression level in ZUKC cohort.

Characteristics	Patients (n=90)	IHC score		χ2	*P* value
		< 6 (%)	>= 6 (%)		
**Age**				4.66	***0.031***
<= 65	65	28 (43.1%)	37 (56.9%)		
> 65	25	4 (16.0%)	21 (84.0%)		
**Gender**				0.15	0.697
Male	60	20 (33.3%)	40 (66.7%)		
Female	30	12 (40.0%)	18 (60.0%)		
**Histologic grade**				4.99	***0.026***
Grade1&2	55	25 (45.5%)	30 (54.5%)		
Grade3&4	35	7 (20.0%)	28 (80.0%)		
**T stage**				5.27	***0.022***
T1-T2	71	30 (42.3%)	41 (57.7%)		
T3-T4	19	2 (10.5%)	17 (89.5%)		
**N stage**				4.58	***0.012***
N0	80	32 (40.0%)	48 (60.0%)		
N1	10	0 (0%)	10 (100.0%)		
**M stage**				0.31	0.416
M0	84	31 (36.9%)	53 (63.1%)		
M1	6	1 (16.7%)	5 (83.3%)		
**Laterality**				0.37	0.544
Left	39	12 (30.8%)	27 (69.2%)		
Right	51	20 (39.2%)	31 (60.8%)		
**Tumor size**				5.75	***0.016***
< 4cm	42	9 (21.4%)	33 (78.6%)		
>= 4cm	48	23 (47.9%)	25 (52.1%)		
**Status**				1.33	0.127
Alive	86	29 (33.7%)	57 (66.3%)		
Dead	4	3 (75.0%)	1 (25.0%)		

Statistical significance was determined by Chi-square test (if necessary, results were adjusted by Yate's correction) or Fisher's exact test (if n less than 5). Bold italics indicate statistically significant values. ZUKC: Zhejiang University Kidney Clear Cell Carcinoma; IHC: Immunohistochemistry.

Supplementary Table S3. Clinicopathological characteristics in relation to APOL1 expression level in the TCGA cohort.

Characteristics	Patients (n=505)	APOL1 expression Low (%)	High (%)	χ2	*P* value
**Age**				3.09	0.079
<= 65	335	158 (47.2%)	177 (52.8%)		
> 65	170	95 (55.9%)	75 (44.1%)		
**Gender**				7.24	***0.007***
Male	333	152 (45.6%)	181 (54.4%)		
Female	172	101 (58.7%)	71 (41.8%)		
**Histologic grade**				21.97	***< 0.001***
Grade1	12	8 (66.7%)	4 (33.3%)		
Grade2	219	127 (58.0%)	92 (42.0%)		
Grade3	201	98 (48.8%)	103 (51.2%)		
Grade4	73	20 (27.4%)	53 (72.6%)		
**Pathologic stage**				23.35	***< 0.001***
Stage I	251	151 (60.2%)	100 (39.8%)		
Stage II	54	23 (41.5%)	31 (58.5%)		
Stage III	119	53 (44.5%)	66 (55.5%)		
Stage IV	81	26 (32.1%)	55 (67.9%)		
**T stage**				17.96	***< 0.001***
T1	257	152 (59.1%)	105 (40.9%)		
T2	65	27 (41.5%)	38 (58.5%)		
T3	172	71 (41.3%)	101 (58.7%)		
T4	11	3 (27.3%)	8 (72.7%)		
**N stage**				5.66	0.06
N0	226	114 (50.4%)	112 (49.6%)		
N1	15	3 (20.0%)	12 (80.0%)		
Nx	264	136 (51.5%)	128 (48.5%)		
**M stage**				13.07	***0.001***
M0	400	211 (52.8%)	189 (47.2%)		
M1	78	25 (32.1%)	53 (67.9%)		
Mx	27	17 (63.0%)	10 (37.0%)		
**OS event**				4.06	***0.044***
None	337	180 (53.4%)	157 (46.5%)		
Yes	168	73 (43.4%)	95 (56.5%)		
**PFS event**				3.79	***0.047***
None	346	184 (53.2%)	162 (46.8%)		
Yes	159	69 (43.4%)	90 (56.6%)		
**DSS event**				5.30	***0.021***
None	397	210 (52.9%)	187 (47.1%)		
Yes	108	43 (39.8%)	65 (60.2%)		

Statistical significance was determined by Chi-square test (if necessary, results were adjusted by Yate's correction) or Fisher's exact test (if n less than 5). Bold italics

indicate statistically significant values. TCGA: The Cancer Genome Atlas; OS: Overall survival; PFS: Progression free survival; DSS: Disease specific survival.

Supplementary Table S4. Clinicopathological characteristics in relation to APOL1 expression level in ZUKC cohort.

Characteristics	Patients (n=90)	IHC score < 6 (%)	IHC score >= 6 (%)	χ2	*P* value
**Age**				4.77	***0.029***
<= 65	65	34 (52.3%)	31 (47.7%)		
> 65	25	6 (24.0%)	19 (76.0%)		
**Gender**				0.95	0.330
Male	60	24 (40.0%)	36 (60.0%)		
Female	30	16 (53.3%)	14 (46.7%)		
**Histologic grade**				4.84	***0.028***
Grade1&2	55	30 (54.5%)	25 (45.5%)		
Grade3&4	35	10 (28.6%)	25 (71.4%)		
**T stage**				4.20	***0.036***
T1-T2	71	36 (50.7%)	35 (49.3%)		
T3-T4	19	4 (21.1%)	15 (78.9%)		
**N stage**				1.72	0.175
N0	80	38 (47.5%)	42 (52.5%)		
N1	10	2 (20.0%)	8 (80.0%)		
**M stage**				0.50	0.401
M0	84	36 (42.9%)	48 (57.1%)		
M1	6	4 (66.7%)	2 (33.3%)		
**Laterality**				1.84	0.175
Left	39	21 (53.8%)	18 (46.2%)		
Right	51	19 (37.3%)	32 (62.7%)		
**Tumor size**				6.15	***0.013***
< 4cm	42	25 (59.5%)	17 (40.5%)		
>= 4cm	48	15 (31.3%)	33 (68.8%)		
**Status**				0.55	0.319
Alive	86	37 (43.0%)	49 (57.0%)		
Dead	4	3 (75.0%)	1 (25.0%)		

Statistical significance was determined by Chi-square test (if necessary, results were adjusted by Yate's correction) or Fisher's exact test (if number less than 5). Bold italics indicate statistically significant values. ZUKC: Zhejiang University Kidney Clear Cell Carcinoma; IHC: Immunohistochemistry.

Supplementary Table S5 Primer sequences, siRNAs and shRNA used in current study.

Primer name		Sequence (5'-3')
NOP2	F	GCATTCTGTACCATGGGGCG
	R	AATCTCCTCTTGGCTGCCCT
ALYREF	F	GCAGGCCAAAACAACTTCCC
	R	AGTTCCTGAATATCGGCGTCT
APOL1	F	GAGGTGAGGGAGTTTTTGGGT
	R	TCGTGTGAGTTGGTAAGTATTGC
APOL1 (for MeRIP)	F	GGGGATAAAGAGGGTGAGGT
	R	CATTCCCCACACTCTCCAGT
RPL14	F	GACCTTGCACTCAAGTGAGGA
	R	CTTGTCGGACATACTTCTGGTG
TRIM8	F	CGTGGAGATCCGAAGGAATGA
	R	CAGGCGCTTGTCTGACTCG
GAPDH	F	GGAGCGAGATCCCTCCAAAAT
	R	GGCTGTTGTCATACTTCTCATGG
β-actin	F	GCAAGCAGGAGTATGACGAG
	R	CAAATAAAGCCATGCCAATC
Control siRNA		UUCUCCGAACGUGUCACGU
		ACGUGACACGUUCGGAGAA
NOP2 siRNA1		GCCUUCCAGAAACAGAAUGAUTT
		AUCAUUCUGUUUCUGGAAGGCTT
NOP2 siRNA2		GCAACGAUCACCUAAAUUATT
		UAAUUUAGGUGAUCGUUGCTT
YBX1 siRNA1		GGCAAUGAAGAAGAUAAAGAAAATT
		UUUUCUUUAUCUUCUUCAUUGCCTT
YBX1 siRNA2		GGAGUUUGAUGUUGUUGAAGGTT
		CCUUCAACAACAUCAAACUCCTT
ALYREF siRNA1		CGUGGAGACAGGUGGGAAATT
		UUUCCCACCUGUCUCCACGTT
ALYREF siRNA2		GGAGUCUCAGACGCCGAUAUUTT
		AAUAUCGGCGUCUGAGACUCCTT
APOL1 siRNA1		GGACAACCUUGCAAGACAATT
		UUGUCUUGCAAGGUUGUCCAG
APOL1 siRNA2		GGAUUACCAGCAGUACCAUTT
		AUGGUACUGCUGGUAAUCCCG
NOP2 shRNA		GCCTTCCAGAAACAGAATGAT
APOL1 shRNA		GGACAACCUUGCAAGACAATT

Supplementary Table S6 Primary antibodies used in current study.

Antigens	Manufacturer	Catalog Number	Application
NOP2	Abcam	Ab271075	1:1000 for WB;<br>1:200 for IHC;<br>1:30 for IP
YBX1	Abcam	Ab76149	1:1000 for WB;<br>1:30 for IP
ALYREF	Abcam	Ab202894	1:2000 for WB
APOL1	Proteintech	11486-2-AP	1:1000 for WB;<br>1:500 for IHC
GAPDH	Fdbio science	FD0063	1:5000 for WB
β-actin	Fdbio science	FD0060	1:5000 for WB
PI3K	ABclonal	A4992	1:1000 for WB
phospho-PI3K (p85)	ABclonal	AP0854	1:1000 for WB
AKT	ABclonal	A17909	1:1000 for WB
phospho-AKT (Ser473)	ABclonal	AP0637	1:1000 for WB
PARP	CST	#9542	1:1000 for WB
Bcl-2	Proteintech	12789-1-AP	1:2000 for WB
BAX	Proteintech	50599-2-Ig	1:2000 for WB
ki67	Proteintech	27309-1-AP	1:2000 for IHC
Anti-5-methylcytosine	Abcam	Ab10805	1:200 for Southern Blot
HRP-Rabbit	Fdbio science	FDR007	1:5000 for WB
HRP-Mouse	Fdbio science	FDM007	1:5000 for WB
